# The association between social class and the impact of treatment for mental health problems: a systematic review and narrative synthesis

**DOI:** 10.1007/s00127-022-02378-9

**Published:** 2022-11-23

**Authors:** Phoebe Barnett, Iyinoluwa Oshinowo, Christopher Cooper, Clare Taylor, Shubulade Smith, Stephen Pilling

**Affiliations:** 1 Centre for Outcomes Research and Effectiveness, Research Department of Clinical, Educational and Health Psychology, University College London, London, WC1E 7HB UK; 2 National Collaborating Centre for Mental Health, Royal College of Psychiatrists, London, UK; 3 Department of Forensic and Neurodevelopmental Science, Institute of Psychiatry, Psychology and Neuroscience, Kings College London, London, UK; 4 South London and Maudsley NHS Foundation Trust, Bethlem Royal Hospital, Beckenham, UK; 5 Camden and Islington NHS Foundation Trust, London, UK

**Keywords:** Systematic review, Social, Mobility, Intervention, Socio-economic status

## Abstract

**Purpose:**

This systematic review aimed to synthesise all quantitative literature on the association between social class and the effectiveness of interventions for mental health disorders.

**Methods:**

Systematic literature searches (inception-March 2021) were conducted across 7 databases, and all quantitative studies meeting inclusion criteria, examining the impact of social class on access to treatment, or intervention effectiveness, or the impact of treatment on social mobility, were synthesised narratively.

**Results:**

Evidence suggests that lower social class may be associated with reduced access to primary and secondary mental health care and increased likelihood of access via crisis services, and patients of lower social class may not benefit from all mental health interventions, with reduced effectiveness. While limited, there was some indication that psychosocial interventions could encourage increased employment rates.

**Conclusion:**

Social class is associated with the effectiveness of psychological interventions, and should be considered when designing new interventions to prevent barriers to access and improve effectiveness.

**Supplementary Information:**

The online version contains supplementary material available at 10.1007/s00127-022-02378-9.

## Introduction

A growing body of literature has suggested that mental health problems are not only associated with distress and impairment, but can also have long term negative consequences on social class and social mobility [1–4]. Social class can be multifaceted, encompassing a number of measures of deprivation, such as income, education and occupational status [5], all of which can be affected. It is likely that this link is reciprocal, with additional research demonstrating good evidence for multiple conceptualizations of social class influencing later mental health [6, 7]. With growing international concern over the rising prevalence of mental health problems to date in both children and adults [8, 9], efforts to provide evidence based, effective interventions have increased [10]. However, the link between mental health and social class extends beyond incidence, and likely also influences treatment outcomes [11], encompassing both symptom severity but also later social mobility [12]. This not only exacerbates impairment and distress, but also contributes to health inequality.

While efforts to establish how indicators of socio-economic status impact mental health treatment have been made [13], relatively little is known about the impact on patients [14]. To date, there has been no review of the literature which considers all interventions in people with both common and severe mental health disorders and their association with social class (both causally and as a result of intervention). In light of this, we aimed to conduct a systematic review, synthesising all quantitative studies of the association between social class and intervention effectiveness for people with mental health disorders, to answer the following questions: (1) Is there an association between social class and access to treatment? (2) Is there an association between social class and effectiveness of interventions for mental health disorders? (3) Do interventions for mental health disorder improve social mobility?

## Methods

This systematic review was undertaken as part of a wider project commissioned by the UK social mobility commission examining the quantitative evidence available exploring the link between diagnosed mental health conditions and social mobility outcomes, and followed PRISMA reporting guidelines [15]. The protocol for this review was not registered on PROSPERO because the review aimed to consider both health and social outcomes, making it ineligible for registration. However, a detailed protocol, defined a priori and followed without variation, is provided in online resource 1.

### Search strategy

Studies were identified using database and non-database search methods [16, 17]. Seven bibliographic databases were searched: MEDLINE (1946-27/09/19), Embase (1980-27/09/19), PsycINFO (1806-September week 2), Health Management Information Consortium (1979-May 2019), Social Policy and Practice (1890-27/09/19), Applied Social Sciences Index Abstracts (01/01/1987-27/09/2019) and Education Resources Information Centre (1966-27/09/19). An update search of all databases was carried out on the 17th March 2021. The following study design literature search filters were used: CADATH RCT/CCT filter [18] and the SIGN filter [19], adapted to focus on studies reporting prospective/retrospective cohort or longitudinal designs. The search was not limited by language or date.

The following supplementary search methods were undertaken: [20]

- Reference lists of systematic reviews meeting inclusion criteria were searched;
- Web-searching was undertaken using google advanced following Briscoe [21].
- The list of studies meeting inclusion were shared with our expert advisory group to identify any studies known to our experts which may not have been identified [16].

Studies were de-duplicated in EndNote X8. The full search strategies are reported in online resource 2.

### Study selection

Studies were independently double-screened by two researchers using Rayyan [22]. A third researcher was available in the event of disagreement.

### Selection criteria

We included randomised controlled trials (RCTs) or cohort studies of populations with a mental health condition. We considered proxies for social class and social mobility, in line with previous research [23]. These were: socio-economic status, employment, income, and education. The following study selection criterion were followed:

1. Reports access to treatment as an outcome and social class (or proxies for social class) subgroups
2. Reports mental health outcomes of an intervention for mental health problems and reports outcomes by social class or examines social class as a predictor.
3. Reports social mobility outcomes (or proxies for social class) for a mental health intervention or treatment.

We excluded studies with substance and alcohol misuse disorders or neurodevelopmental disorders as the study population. We also excluded dissertations, conference abstracts and protocols.

### Quality appraisal

Quality appraisal was undertaken by one reviewer and checked by another. The Cochrane Risk of Bias (ROB) tool was used for RCTs [24] and a version of the Newcastle–Ottawa (NOS) tool modified by Gondek et al. was used for cohort studies [23]. In line with recommendations [24], We characterised RCTs according to aspects particularly relevant for our research questions. Therefore, we classified RCTs as high ROB if either comparability of groups or attrition were rated as high ROB, unclear if either of these aspects was rated as unclear (with the other rated as low ROB) and low ROB where both these aspects were low ROB. Cohort studies were considered as “good”, “fair” and “poor” quality when they scored seven, six, or five or less on the modified NOS tool respectively.

### Data extraction

A data extraction tool was developed by the research team and piloted. Data extraction was undertaken by one reviewer and checked by another. The following criteria was extracted: study design, country, and region of study, setting, mental health condition and population characteristics, study purpose, intervention/exposure, comparison/control, social mobility outcome measured and method of measurement and limitations.

### Synthesis of data

We synthesised the results narratively [25]. We organised studies into categories according to research question, study type, mental health condition and social class outcome, and produced summary tables of findings. We did not carry out a meta-analysis due to the heterogeneity of populations, interventions, outcomes, and adjustments made to reported analyses.

## Results

**Fig. 1:**
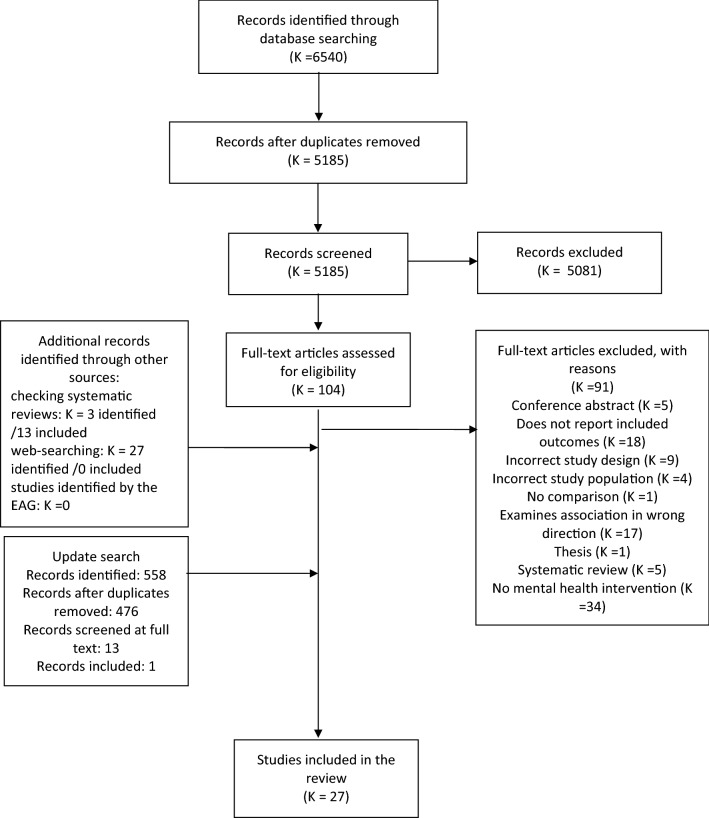
PRISMA Diagram. Included studies were carried out in a variety of countries (*K* = 12 USA, *K* = 6 UK, *K* = 2 Sweden, *K* = 2 Finland, *K* = 1 Australia, *K* = 1 Denmark, *K* = 1 Germany, *K* = 1 Norway, *K* = 1 multiple). The quality of the evidence was mixed, with seven studies being rated as “good quality” or “low ROB”, six studies rated as “fair quality” or “unclear ROB” and 14 studies rated as “poor quality” or “high ROB”. Table 1 shows the characteristics of each included study

In total, 4792 studies were identified by the bibliographic and non-bibliographic searches after de-duplication. From 101 potentially relevant studies assessed at full text, 13 studies were included in the review. An additional study was found during an update search conducted March 2021. Three systematic reviews were searched for additional studies for inclusion. Thirteen of the final 27 studies were discovered through this method. The full systematic search process is shown in Fig. 1.

**Table 1:** Characteristics of included studies

Study reference	Country (region)	Sample size	Intervention Setting	Study design	Intervention/exposure	Comparison	Mean age (range)	Gender (% female)	Ethnicity	Follow-up	Study quality
Cummings [26]	USA	1133	Clinic	Prospective cohort	County-level SES	NA	15.8 (NR)	59.2	White: 38.9% Hispanic: 25.6% Black: 24.0% Asian: 9.6% Other: 1.9%	1 year	Good quality (6)
Dorner [27]	Sweden (Stockholm)	66,097	Inpatient	Prospective cohort	Educational level	NA	NR (18–59)	69.2	Country of birth: Sweden (86.3%) other Northern European (3.2%) rest of world (8.4%)	4 years	Good quality (7)
Paananen et al. [28]	Finland	59,476	Specialised psychiatric care	Prospective cohort	Parental socio-economic background Parental educational attainment	NA	NR (0–21)	NR	NR	21 years	Poor Quality (6)
Packness et al. [29]	Denmark	50,374	Mental health care services	Prospective Cohort	Income Education level	NA	NR (20–64)	56.9	Country of birth: Denmark: 84.40% European and western countries: 8.21% Non-western countries and unknown: 7.38%	12 months	Poor Quality (5)
Shah et al. [30]	UK (England and Wales)	17,197	Clinic-general practice	Prospective cohort	Social Class	NA	NR	NR	NR	11 months	Poor quality (3)
Bennett et al. [31]	USA	404	Community	RCT-secondary analysis	NAVIGATE (coordinated speciality care for schizophrenia)	Usual community services	23 (15–40)	27.5	White: 54% African American: 37.6% Other: 8.4% Hispanic: 18.1%	End of treatment	High risk of bias
Button et al. [32]	UK	297	NA	RCT—secondary analysis	Online CBT	Waitlist control	34.95 (18–75)	68.01	NR	4 months	High risk of bias
Cort et al. [33]	USA (New York)	70	Community	RCT—secondary analysis	Standardized interpersonal psychotherapy (IPT)	Usual care psychotherapy	36 (NR)	100	White: 59% Black: 41%	2 years	High risk of bias
El Alaoui et al. [35]	Sweden (Stockholm)	764	Psychiatric clinic	Prospective cohort	Internet cognitive behavioural therapy (ICBT)	Pre-ICBT	32.51(NR)	46.0	NR	End of treatment	Fair quality (5)
Delgadillo et al. [34]	UK	28,498	Community	Retrospective case note review	IAPT	Pre treatment	38.27 (16–92)	64.6	White: 85.5%	End of treatment	Good quality (6)
Falconnier et al. [36]	USA (Washington, Pennsylvania, Oklahoma)	239	Hospital	RCT	Psychotherapy or pharmacotherapy	Placebo plus clinical management	NR (21–60)	70	White: 89% African American: 9% Hispanic: 2% Other non-white: less than 1%	6, 12, 18 months	Low risk of bias
Fournier et al. [37]	USA (Philadelphia)	180	Outpatient	RCT	CBT	Placebo	39.94 (NR)	NR	Caucasian: 83%	16 weeks	Unclear risk of bias
Gift et al. [38]	USA	217	Inpatient	Prospective cohort	Inpatient admission	Pre inpatient admission	NR (15–55)	NR	NR	2 years	Poor quality (3)
Gilman et al. [39]	USA (New York, Philadelphia, Pittsburgh)	514	Primary care	RCT	guideline based provision of depression treatment (citalopram or psychotherapy)	Usual care	NR	72	NR	2 years	Unclear risk of bias
Hoyer et al. [40]	Germany (Bochum, Dresden, Göttingen, Jena and Mainz)	237	Outpatient	Before and after trial	CBT	Pre-CBT	34.94 (18–70)	55.2	NR	15 weeks	Good quality (6)
Joutsenniemi et al. [41]	Finland (Helsinki)	326	Outpatient	RCT	Long term therapy	Short term therapy	NR	75.8	NR	3 years	High risk of bias
Kelly et al. [42]	USA (New Haven)	1004	Primary care	RCT-secondary analysis	Collaborative care (CALM) or usual care	NA	NR (18–75)	70.0	NR	6 months	Low risk of bias
Kodal et al. [11]	Norway	179	Community mental health clinics	RCT	Individual CBT	Group CBT	15.5 (NR)	54.7	NR	End of treatment, 12 months	Unclear risk of bias
Myers et al. [43]	USA (New Haven)	1565	Inpatient	Retrospective cohort	Psychiatric treatment	NA	NR	NR	NR	10 years	Poor quality (5)
Pirkis et al. [44]	Australia	16,700	Community	Retrospective service evaluation	Access to Allied Health Professionals (ATAPS)	Pre treatment	NR	73.1	NR	NR	Fair quality (4)
Poots et al. [45]	UK (London)	6062	Community (IAPT)	Retrospective service evaluation	Westminster IAPT services	Pre IAPT use	NR	NR	NR	3 years, 3 months	Poor quality (4)
Tohen et al. [46]	USA (Boston)	75	Hospital (inpatient)	Prospective cohort	48 months post hospital	6 months post hospital	NR (17-NR)	NR	White: 97.3%	4 years	Fair quality (4)
Dion et al. [47]	USA (Massachusetts, Belmont)	67	Hospital (inpatient)	Prospective cohort	Hospital admission	Pre hospital admission	31.4 (17–59)	68.0	White: 97.77% Oriental: 3.33%	1 year	Poor quality (5)
Kozma et al. [48]	Multiple (South-Africa (Cape Town) and USA (South Carolina))	1012	Outpatient	Open label trial extension	Paliperidone extended-release	Pre-treatment	37.7 (NR)	40.9	NR	52 weeks	Poor quality (4)
Perry et al. [49]	UK (North-west England)	69	Community	Single blind RCT	Teaching patients with bipolar disorder to identify early symptoms of relapse and seek prompt treatment from health services, plus routine care	Routine care alone	44.51 (NR)	68.1	White: 91.30%	6, 12, 18 months	Low risk of bias
Roy Chengappa et al. [50]	USA (Pittsburgh)	139	Hospital	Open label trial extension	Olanzapine	Pre-treatment	39.5 (NR)	48.0	NR	1 year	Poor quality (4)
Tsiachristas et al. [12]	UK (Oxford)	3674	Community	Retrospective cohort	Early intervention in psychosis	Other community mental health teams	27.13 (NR)	42.5	White British: 37.21%	3 years	Poor quality (6)

### The impact of social class on access to treatment

Five studies examined the relationship between social class and access to treatment [26–30]. These examined different points of access on the care pathway for depressive disorders (*K* = 2) and mixed mental health disorders (anxiety and depressive disorders) (*K* = 3). Two studies looked at the intergenerational impact of parental social class on access to treatment. These examined access to treatment during adolescence, and assumed that the social class of adolescents was the same as that of their parents. Three studies examined the intragenerational impact of social class on access to mental health treatment. There were no data on employment or social class, or social mobility itself. Table 2 provides a summary of findings.

**Table 2:** The impact of social class on access to treatment

Predictor	Intragenerational/intergenerational	Mental health disorder^a^	Outcome (access to treatment)	Age	Study (quality)	Findings
Educational attainment	Intergenerational	Any common mental health disorder	Access to specialist psychiatric services	Adolescence	PAANANEN2013 (Poor quality)	The use of specialised psychiatric care was significantly more common among children of parents with a short education (males: OR: 2.03, 95% CI 1.78,2.32, *p* < 0.001; females: OR: 1.93, 95% CI 1.71, 2.18, *p* < 0.001) than those with a long education. A short education (OR: 3.96, 95% CI 2.65, 5.93, *p* < 0.001) strongly determined the use of psychiatric inpatient care before 13 years, especially among females (no data reported)
County-level socio-economic status		Depressive Disorders	Clinical counselling	Adolescence	CUMMINGS2014 (Good quality)	There were significantly higher odds of use of counselling in a clinical setting in places of higher county-level affluence (OR 1.35, 95% CI 1.10, 1.66, *p* = 0.004) when controlling for county-level racial/ethnic composition. There were significantly lower odds of use of counselling in a clinical setting in places of county-level disadvantage after adjusting for county racial/ethnic composition (OR: 0.66, 95% CI 0.47, 0.92, *p* = 0.015)
Socio-economic status		Any common mental health disorder	Access to specialist psychiatric services	Adolescence	PAANANEN2013 (Poor quality)	The use of specialised psychiatric care was significantly more common among children of parents with low SES (males: OR: 1.58, 95% CI 1.44, 1.73, *p* < 0.001; females: OR: 1.55, 95% CI 1.43,1.69, *p* < 0.001) than those with parents with high SES Parental low SES (OR 2.75, 95% CI 2.03,3.73, *p* < 0.001) strongly determined the use of psychiatric inpatient care before 13 years, especially among females (no data reported)
Educational attainment	Intragenerational	Depressive disorders	Mental health treatment	Adulthood	PACKNESS2017 (Poor quality)	Contact with a psychologist was less likely for those with fewer years of education (OR: 0.37; 95% CI 0.35, 0.40, *p* < 0.05) compared with higher educational groups. There was less use of GP mental health services in lower educational groups (OR: 0.71; 95% CI 0.67, 0.75, *p* < 0.05) compared with higher educational groups. In people who did have contact, those who had lower education had lower rates of visits to outpatient psychiatrists (IRR: 0.75, 95% CI 0.74, 0.76, *p* < 0.05), psychologists (IRR: 0.80, 95% CI 0.79, 0.82, *p* < 0.05) and visits to GP mental health services (IRR: 0.93, 95% CI 0.91, 0.96, *p* < 0.05) compared with those with higher education
		Any common mental health disorder	Future inpatient care	Adulthood	DORNER2017 (Good quality)	Individuals with low educational attainment had a higher proportion of specialised health care than individuals with higher education. Combined psychiatric medication and medication with anxiolytics was more common among individuals with low educational attainment. There was a significant interaction between educational attainment and applied medication regimes: this predicted subsequent inpatient care for mental health problems (*p* = 0.007) and subsequent suicide attempts (*p* = 0.026). Higher educational attainment resulted in a stronger association between medication regime and future inpatient care
Income		depressive disorders	Mental health treatment	Adulthood	PACKNESS2017 (Poor quality)	People with the lowest incomes established contact with outpatient psychiatrists more often (OR: 1.25; 95% CI 1.17, 1.34, *p* < 0.05) compared with people in the highest income group. Contact with a psychologist was less frequent for lower income groups (OR: 0.49; 95% CI 0.46, 0.53, *p* < 0.05) compared with higher income groups. Lower income groups used GP mental health service less frequently (OR: 0.81; 95% CI 0.77, 0.86, *p* < 0.05) compared with higher income groups. There was no significant association between income and emergency service contact. In people who did have contact, those who had a lower income had lower rates of visits to outpatient psychiatrists (IRR: 0.83, 95% CI 0.81, 0.84, *p* < 0.05), psychologists (IRR: 0.94, 95% CI 0.91, 0.96, *p* < 0.05) and GP mental health services (IRR: 0.94, 95% CI 0.92, 0.97, *p* < 0.05) compared with those with higher income, when adjusted for socio-demographics, comorbidity and access to a vehicle
Socio-economic status		Any common mental health disorder	Consultations	Elderly	SHAH2001 (Poor quality)	For all mental health problems, rates of consultation were highest among people from social class V, but overall there was no consistent association between social class and consultation rates. This was also the case for the consultation rates for each diagnostic group

^a^Anxiety or depressive disorder

#### Educational attainment

A good quality study [27] found that in adults with mixed CMDs, having a low educational level predicted more use of specialised health care, and more prescription of combined psychiatric medication, and medication with anxiolytics, compared to people with higher educational attainment (*p* < 0.001). There was a significant interaction between education level and applied medication regime, such that having a lower educational attainment predicted a weaker association between the regime prescribed and chances of future inpatient care. In people of higher educational attainment, the chosen regime was more important in determining outcomes (*p* = 0.007). The interaction between educational attainment and medication regime also significantly predicted chances of attempted suicide (*p* = 0.026). A poor quality study [29] suggested that having a low educational attainment reduces contact with GPs (OR = 0.71, (95%) CI 0.67–0.75, *p* < 0.05) and psychologists (OR = 0.37, CI 0.35–0.40, *p* < 0.05). Another poor quality study [28] suggested that there was an increased use of inpatient care before age 13 (OR = 3.96, CI 2.65–5.93, *p* < 0.001) and increased specialist service use (males: OR = 2.03, CI 1.78–2.32, *p* < 0.001; females: OR = 1.93, CI 1.71–2.18, *p* < 0.001) in those with low parental education.

#### Income

A study of poor quality [29] found that low family income predicted reduced odds of contact with psychologists (OR = 0.49, CI 0.46–0.53, *p* < 0.05) and GP health services (OR = 0.81, CI:0.77–0.86, *p* < 0.05) compared to those with higher income and reduced rates of visits in those who did have contact (visits to outpatient psychiatrists (Incidence Rate Ratio (IRR) = 0.83, CI 0.81–0.84, *p* < 0.05), psychologists (IRR = 0.94, CI 0.91–0.96, *p* < 0.05) and visits to GP mental health services (IRR = 0.94, CI: 0.92–0.97, *p* < 0.05)).

#### Socio-economic status

One good quality study [26] found that adolescents with depression living in more affluent areas had significantly higher odds of accessing counselling services (OR = 1.35, CI 1.10–1.66, *p* = 0.004). A study [30] of poor quality found that the association between socio-economic status and consultation rates for psychiatric disorders was weak in older people with mixed CMDs. Although rates were highest among older people from social class V, overall there was no association. Another study [28] of poor quality found that low parental socio-economic status (SES) predicted more use of specialised psychiatric care among children (males: OR = 1.58, CI 1.44–1.73, *p* < 0.001; females: OR = 1.55, CI 1.43–1.69, *p* < 0.001) than those with high parental SES. Low parental SES also strongly predicted psychiatric inpatient care use before the age of 13 (OR = 2.75, CI 2.03–3.73, *p* < 0.0001).

#### Summary: the association between social class and access to mental health treatment

Evidence for the association between social class and access to mental health treatment is limited and of varying quality but suggests that lower social class is associated with reduced access to primary and secondary mental health care and an increased likelihood of accessing crisis services, such as inpatient admission, which may be independent of earlier intervention.

### The association between social class and mental health outcomes following treatment

Seventeen studies examined the relationship between social class (and indicators relating to social class) and mental health treatment outcomes [11, 31–46]. These included randomized controlled trials (RCTs) (*K* = 9), a before and after trial (*K* = 1) and cohort studies (*K* = 7). Outcomes of treatment for anxiety disorders (RCT: *K* = 2, Cohort: *K* = 1, pre-post: *K* = 1), depressive disorders (RCT: *K* = 5, Cohort: *K* = 1), mixed anxiety and depressive disorders (RCT: *K* = 1, Cohort: *K* = 2), psychosis (RCT: *K* = 1, Cohort: *K* = 2) and bipolar disorder (Cohort: *K* = 1) were reported. One study examined the impact of family social class on adolescent mental health treatment outcomes, while another examined the impact of family occupational status on adolescent treatment outcomes. These assumed that social class in adolescents is the same as that of their parents. All remaining studies examined social class intragenerationally, such that a person’s own social class and intervention outcomes were examined. Table 3 provides a summary of findings.

**Table 3:** The association between social class and mental health outcomes following treatment

Social Mobility predictor	Study Design	Severity	Mental health disorder	Outcome	Age	Study (quality)	Treatment	Findings
Educational attainment	RCT	CMD^a^	Depressive disorders	Symptom severity	Adulthood	FALCONNIER2009 (Low risk of bias)	Psychotherapy or pharmacotherapy	Educational attainment (having more or less education than a secondary school qualification) was not a significant predictor of outcome when controlling for other covariates when symptom severity was measured on the HRSD (b: 0.56, SE: 0.40, *p* = 0.155), BDI (b: 0.13, SE: 0.63, *p* = 0.833) or Global Assessment Scale (GAS) (b: − 0.13, SE: 0.61, *p* = 0.063)
			Depressive disorders	Symptom severity	Adulthood	BUTTON2012 (High risk of bias)	Online CBT	There was no evidence of an interaction between educational attainment and treatment. Less than ‘A’ level (compared with ‘A’ level or above) b: − 2.9 95% CI − 9.3, 3.5, *p* = 0.372
			Any common mental health disorder	Symptom severity	Adulthood	JOUTSENNIEMI2012 (High risk of bias)	Long term psychotherapy and short term treatment	A university education or a basic education predicted a sufficient outcome for short-term treatment, whereas an intermediate education predicted the need for long-term psychotherapy
	non-RCT	CMD	Social anxiety	Symptom severity	Adulthood	HOYER2016 (Good quality)	CBT	Controlling for baseline Liebowitz Social Anxiety Scale score, age, gender and educational attainment did not predict improvements in symptoms at end of treatment
			Any common mental health disorder	Symptom severity	Adulthood	PIRKIS2011 (Fair quality)	Access to allied health professionals (ATAPS) ^b^	Having any level of education predicted more treatment gains than not completing secondary school: completed education to year 10: b:1.50 95% CI 0.49, 2.51, *p* = 0.004; completed education to year 11: b:1.36 95% CI 0.57, 2.15, *p* = 0.001; completed education to year 12: b:1.42, 95% CI 0.49, 2.35, *p* = 0.003; completed tertiary education: b:1.58, 95% CI 0.61, 2.55, *p* = 0.002
Occupational status	RCT	CMD	Depressive disorders	Symptom severity	Adulthood	CORT2012 (High risk of bias)	Standardized interpersonal psychotherapy	Occupational status was significantly associated with improvement on the HRSD: employed (versus unemployed) b: − 3.58 SE:1.32 95% CI − 6.7, − 0.98, *p* = 0.02
			Depressive disorders	Symptom severity	Adulthood	FOURNIER2009 (Unclear risk of bias)	CBT	For participants who were employed, there was no difference between the two treatments (t[155] = -0.67, Cohen’s d = − 0.12, 95% CI − 0.47, 0.23, *p* = 0.51); however, for unemployed participants, cognitive therapy was associated with superior outcomes relative to medication (t[163] = 3.24, Cohen’s *d* = 1.19, 95% CI 0.41, 1.97, *p* = 0.002)
			Any common mental health disorder	Symptom severity	Adulthood	JOUTSENNIEMI2012 (High risk of bias)	Long term psychotherapy and short term treatment	Employed people benefited more from long-term psychotherapy, whereas short-term treatment was sufficient for the rest, with the exception of homemakers, who received no help from either therapy for general symptoms or anxiety symptoms
		CMD	Any common mental health disorder	Symptom severity	Adulthood	DELGADILLO2017 (Good quality)	IAPT	People who were unemployed had more severe symptom measures following IAPT treatment. Unemployed versus employed: PHQ-9 b: 0.68, SE: 0.07, *p* < 0.001; GAD 7 b: 0.54, SE: 0.07, *p* < 0.001
			Social anxiety	Symptom severity	Adulthood	EL ALAOUI2015 (Fair quality)	Internet CBT	Controlling for age, global functioning, adherence and treatment credibility rating, being employed (versus unemployed) predicted significantly lower social anxiety symptoms at follow-up (b:− 2.29 SE: 0.95, *p* < 0.05)
		SMI^c^	Bipolar	Relapse	Adulthood	TOHEN1990 (Fair quality)	Naturalistic treatment (clinician decided)	Poor occupational status at baseline did not significantly predict relapse 48 months post-treatment (HR: 1.1, SE: 0.34, *p* > 0.05)
Income	RCT	CMD	Depressive disorders	Symptom severity	Adulthood	FALCONNIER2009 (Low risk of bias)	Psychotherapy or pharmacotherapy	Controlling for social functioning, cognitive dysfunction, expectation of improvement, endogenous depression, duration of current episode and age, family income predicts depression measured using the BDI, but not the HRSD (self-rated). The percent of variance explained by family income was only 1%. HRSD: 0.0% variance explained b:− 0.03, SE: 0.06, *p* = 0.559. BDI: 0.9% variance explained b:− 0.22 SE: 0.09, *p* = 0.016*
			Anxiety disorders			KELLY2015 (Low risk of bias)	Collaborative care or usual care	After controlling for intervention assignment, baseline severity, satisfaction, diagnosis, previous use of CBT, having a lack of money predicted lower odds of remission at 6 months (OR: 0.72, 95% CI 0.56, 0.93, *p* = 0.019)
			Depressive disorders			GILMAN2013 (Unclear risk of bias)	Care managers assigned to ensure guideline based provision of depression treatment (citalopram or psychotherapy)	The intervention was more effective among participants under conditions of financial strain, both between baseline and 4 months (intervention mean reduction 5.9, 95% CI: − 11, − 0.8 for participants under financial strain; 2.9, 95% CI: − 4.8, − 0.9 for participants without financial strain). Averaged across all follow-ups, the difference in intervention effect between participants under financial strain and participants without financial strain was − 4.5 (95% CI − 0.86, − 0.3)
Social Class	RCT	CMD	Anxiety disorders	Loss of anxiety diagnosis	Adolescence	KODAL2018 (Unclear risk of bias)	Individual CBT	Low family social class was negatively associated with no longer meeting diagnostic criteria for any anxiety related disorder at 2-year follow-up (OR: 0.07, 95% CI 0.01, 0.55, *p* = 0.03), and loss of principal inclusion anxiety diagnosis at 2-year follow-up (OR: 0.26, 95% CI 0.09, 0.75, *p* = 0.04). No other parent-related predictors were associated with long-term changes in youth anxiety
			Depressive disorders	Symptom severity	Older adults	GILMAN2013 (Unclear risk of bias)	Individual CBT	The intervention was equally effective across groups irrespective of level of census-tract poverty, both between baseline and 4 months (intervention mean reduction -3.5 high poverty, -3.2, SE:1.1, SE:1.7 low poverty). Averaged across all follow-ups, the difference in intervention effect between high and low poverty was 0.9 (95% CI − 2.1, 3.9, *p* > 0.05)
					Adulthood	FALCONNIER2009 (Low risk of bias)	Psychotherapy or pharmacotherapy	People from classes IV and V (working class and poor) showed less improvement following psychotherapy or pharmacotherapy than those from classes II and III (‘middle class’) on the HRSD (b:0.96, SE:0.37, *p* = 0.011), explaining 2.8% of variance is symptom severity. However, depression measured on the BDI was not predicted by indicators of social position (b:1.13, SE:0.61, *p* = 0.065). People from class I did not differ in improvement from people from ‘middle classes’
	non-RCT	CMD	Any common mental health disorder	Symptom severity	Adulthood	DELGADILLO2017 (Good quality)	IAPT	People from areas of higher deprivation had higher symptom severity measures following treatment in IAPT services IMD (reference category 1st IMD quintile) b (SE) 2nd quintile PHQ-9 b:− 0.77 SE: 0.11, *p* < 0.001 GAD 7: b: − 0.63SE: 0.10, *p* < 0.001 3rd quintile PHQ-9 b:− 1.11, SE: 0.11, *p* < 0.001 GAD 7: b: − 0.85 SE: 0.09, *p* < 0.001 4th quintile PHQ-9: b:− 1.36 SE: 0.11, *p* < 0.001 GAD 7: b: − 1.03, SE: 0.10, *p* < 0.001 5th quintile PHQ-9 b: − 1.75 SE: 0.12, *p* < 0.001 GAD 7: v: − 1.33 SE: 0.10, *p* < 0.001
			Depressive disorders			POOTS2014 (Poor quality)	IAPT	IAPT services from areas of different levels of deprivation did not show different changes on the PHQ-9 according to IMD category (F(2, 1,417) = 0.90, *p* = 0.406)
		SMI	Psychosis	Symptom severity	Adulthood	GIFT1986 (Poor quality)	Psychiatric treatment	Individual social class was significantly associated with symptom severity at follow-up (*r* = − 0.12). Parental social class was significantly associated with symptom severity at follow-up (*r* = 0.25)
			Psychosis	Treatment status	Adulthood	MYERS1965 (Poor quality)	Psychiatric treatment	Significantly more people from ‘lower classes’ who were having treatment for psychosis were still hospitalised 10 years later (39% class I–II, 49% class III, 52% class IV, 57% class V). More people from higher classes were living in the community at follow-up (30% classes I and II, 27% class III, 18% class IV, 10% class V)
Socio-economic status	Non-RCT	SMI	Bipolar	Relapse	Adulthood	TOHEN1990 (Fair quality)	Naturalistic treatment (clinician decided)	Lower SES at baseline did not significantly predict relapse 48 months post-treatment (HR: 0.7 SE: 0.47, *p* > 0.05)
	RCT	SMI	Schizophrenia	Symptom severity	Adulthood	BENNETT2020 (High risk of bias)	Coordinated speciality care for schizophrenia	NAVIGATE reduced psychotic symptoms by 0.45 standard deviations (*P* = 0.002) for patients in the highest SES quartile. However, in SES quartile 1, the program increased PANSS measures by 0.12 standard deviations. Overall, the differential impact of SES on outcomes was significant (*P* = 0.02)

^a^Anxiety or depressive disorders

^b^Intervention to improve access to low intensity treatment, similar to IAPT

^c^Psychosis and bipolar disorder

#### Common mental disorders (CMDs)

##### Social class

*RCT evidence* Three RCTs examined how social class impacted on treatment for CMDs. One low ROB RCT in adults with depression [36] found that patients from lower social classes (classes IV and V) who were treated with either psychotherapy or pharmacotherapy had lower rates of improvement measured on the Hamilton Rating Scale for Depression (*b* = 0.96, SE = 0.37, *p* = 0.011) than people from classes III and II. However, outcomes measured on the Beck Depression Inventory were not predicted by indicators of social class (*b* = 1.13, SE = 0.61, *p* = 0.065). There was no significant difference between people from Class I and people from Classes II and III. Another study of unclear ROB [11] found that in adolescents with an anxiety disorder treated with individual CBT, family social class was negatively associated with no longer meeting diagnostic criteria for any anxiety-related disorder at 2-year follow up (OR = 0.07, CI 0.01–0.55, *p* = 0.03). Also, lower family social class was negatively associated with loss of principal inclusion anxiety diagnosis at 2-year follow up (OR = 0.26, CI 0.09–0.75, *p* = 0.04). Another RCT of unclear ROB [39] found that assigning a care manager to ensure guideline-based provision of treatment for older adults with depression was equally effective across groups irrespective of area-level deprivation (averaged across all follow ups, the difference in intervention effect between high and low poverty areas was not significant (0.9, CI − 2.1 to 3.9, *p* > 0.05).

*Non-RCT evidence* One study of good quality [34] found that patients from areas of higher deprivation had worse outcomes following treatment in IAPT services (*p* < 0.001). However, a study of poor quality [45] found that IAPT outcomes did not differ in different levels of deprivation. There was no significant effect of Index of Multiple Deprivation (IMD) category on average change in PHQ9 items (F(2, 1417) = 0.90, *p* = 0.406).

#### Occupational status

*RCT evidence* Three RCTs reported how occupational status was associated with treatment effectiveness. An RCT of unclear ROB [37] found that in adults with depression, occupational status predicted whether cognitive therapy was more effective than medication: in employed participants, there was no difference in treatment outcomes (t(155) = − 0.67, *p* = 0.51, Cohen’s d = − 0.12, CI − 0.47 to 0.23), but unemployed participants showed more symptom reduction when treated with cognitive therapy relative to medication (t(163) = 3.24, *p* = 0.002, Cohen’s d = 1.19, CI 0.41–0.97). Two high ROB RCTs found that occupational status in adults had an impact on which treatments (longer or shorter term psychotherapy) were effective for mood and anxiety disorders [41] and on the effectiveness of interpersonal psychotherapy [33] in reducing symptoms of depression: employed (vs unemployed) *b* = − 3.58 SE = 1.32 CI− 6.7 to 0.98, *p* = 0.02.

*Non-RCT evidence* A good quality cohort study [34] found that unemployed people had worse outcomes following treatment in IAPT services (*b* = 0.54–0.68, SE: 0.07, *p* < 0.001). A fair quality cohort study [35] also found that in adults with anxiety disorders, being employed predicted better outcome of CBT delivered online at end of treatment (*b* = − 2.29 SE = 0.95, *p* < 0.05).

#### Educational attainment

*RCT evidence* Three RCTs examined the relationship between education level and effectiveness of interventions in reducing symptoms of CMDs. A low ROB RCT [36] found that in adults with depression given either psychotherapy or pharmacotherapy, education (having more or less education than having a secondary school qualification) was not a significant predictor of outcome when controlling for other covariates (HRSD: *b* = 0.56, SE = 0.40, *p* = 0.155, BDI: *b* = 0.13, SE = 0.63, *p* = 0.833). Two other RCTs of high ROB found that educational attainment did not moderate treatment outcome following online CBT for adults with depression [32] at 4 month follow up: (less than A-level education *b* = − 2.9, CI − 9.3 to 3.5, *p* = 0.372) but that educational attainment may predict how long interpersonal psychotherapy treatment needs to be to have positive outcomes [41].

*Non-RCT evidence* One study of good quality [40] found that the educational attainment of adults with anxiety disorders did not predict improvements in symptoms at end of CBT treatment. A fair quality study [35] found that in adults given an intervention to improve access to treatment, people with higher educational attainment made more treatment gains. Those who had completed secondary school experienced improvements of 1.36–1.58 points higher than those who had not (*p* = 0.001–0.004).

*RCT evidence* Three RCTs reported how income was associated with treatment effectiveness. One RCT of low ROB [36] found that, measured on the BDI, family income explained 1% of the variance in depressive symptoms at end of CBT treatment (*b* = − 0.22, SE = 0.09, *p* = 0.016). However, variance in symptoms measured on the HRSD (*b* = − 0.03, SE = 0.06, *p* = 0.559) was not explained by family income. Another low ROB RCT [42] showed that having a perceived ‘lack of money’ predicted lower odds of remission at 6 months post-treatment for an anxiety disorder (OR = 0.72, CI 0.56–0.93, *p* = 0.019). However, an RCT of unclear ROB [39] found that more ‘financially strained’ older adults with depression consistently improved on symptom measures following guideline-based treatment (citalopram or psychotherapy) more than those who were less financially strained. Averaged across all follow-ups, the difference in intervention effect between financially strained and not was − 4.5, (CI − 8.6–0.3, *p* < 0.05).

### Severe mental illness (SMIs)

#### Social class

*Non-RCT evidence* Two poor quality cohort studies examined the differential impact of social class on intervention effectiveness in adults with psychosis. One study [43] found a significant relationship between social class and follow-up treatment status, such that more people of lower social class remained hospitalised 10 years later (39% class I–II, 49% class III, 52% class IV, 57% class V). Another [38] found that both individual (*r* =− 0.12) and parental social class (*r* = − 0.25) were significantly associated with symptom severity at follow-up after treatment, with lower social class being associated with more severe symptoms. However, both of these studies were published over 22 years ago (1965 and 1986, respectively so results may be less representative of current conceptualisations of social class*.*

#### Socio-economic status

*RCT evidence* A high ROB RCT study [31] of patients with schizophrenia found that socio-economic status had some impact on psychotic symptoms over the course of the two-year intervention (*p* = 0.02)- the interventions effect in reducing psychotic symptoms was less pronounced in the first quartile (lower SES) groups.

*Non-RCT evidence* A fair quality cohort study [46] of people with bipolar disorder found that socio-economic status did not predict relapse following treatment 48 months post-treatment (Hazard ratio (HR) = 0.7, SE = 0.47). However, this study was published in 1990 and therefore should be considered with caution.

#### Occupational status

*Non-RCT evidence* The same study [46] found that people were not more or less likely to relapse according to their occupational status 48 months post-treatment (HR = 1.1, SE = 0.34).

#### Summary: CMDs

Low social class may be associated with poorer treatment outcomes in people with CMDs, hindering improvement following intervention. Occupational status may also play some role in influencing the effectiveness of mental health interventions and some interventions may need to be adapted to be of benefit to people of a lower educational attainment. Tailored care (having access to psychosocial interventions as well as, or instead of, medication) may limit the impact of deprivation on reducing intervention effectiveness, for example, for people with lower educational attainment or income, or people who are unemployed.

#### Summary: SMIs

Evidence regarding the relationship between proxies for social class and treatment for SMIs is extremely limited and in many cases, outdated. There is some suggestion of no relationship between social class and treatment outcomes in people with bipolar disorder, though social class may play a role in psychosis. This possible relationship should be further explored.

### Summary: the association between social class and treatment outcomes

Overall, evidence from interventions for CMDs suggests that people of lower social class may not gain as much benefit from mental health interventions as those of higher social class. Tailoring interventions (so that people with lower educational attainment and income or those who are unemployed have access to psychosocial interventions as well as, or instead of, medication) may help to reduce the impact of these variables on intervention effectiveness. Evidence is limited on the association between social class and intervention effectiveness in people with SMI.

### The association between treatment and social class outcomes following treatment

Six studies examined the relationship between being treated for a mental health disorder and social mobility [12, 46–50]. These included one RCT and five cohort studies. Two cohort studies examined employment outcomes for patients with schizophrenia (*K* = 1) and bipolar disorder (*K* = 1) following pharmacological intervention. One RCT and three Cohort studies examined employment outcomes (RCT: *K* = 1, Cohort: *K* = 2) and education outcomes (Cohort: *K* = 1) for bipolar disorder (RCT: *K* = 1, Cohort: *K* = 2) and psychosis unspecified (Cohort: *K* = 1). All studies examined social mobility outcomes intragenerationally: changes in social status following treatment compared to prior social status. Table 4 provides a summary of the quantitative findings of the identified studies.

**Table 4:** The association between treatment and social class outcomes following treatment

Intervention type	Outcome	Study Design	Mental health disorder	Study (quality)	Treatment	Findings
Pharmacological	Occupational status	Cohort	Schizophrenia	KOZMA2011 (Poor quality)	Paliperidone extended-release	The percentage of people in full-time competitive employment increased from pre-treatment by 81.6% after 52 weeks following treatment (a change from 4.8% of the sample to 8.8% of the sample, *p* < 0.0001). There was a 114% increase in the percentage of people who were in either full- or part-time competitive employment (*p* < 0.0001) and an 88% increase in people who were in any employment (*p* < 0.0001)
			Bipolar disorder	ROY CHENGAPPA2005 (Poor quality)	Olanzapine	Treatment with olanzapine did not improve rates of paid employment: Pre-treatment paid employment: 64/107 (59.81%) Post-treatment paid employment pay: 35/113 (30.97%) OR of employment after treatment: 0.30, 95% CI 0.17, 0.53, *p* < 0.0001
Psychological	Occupational status	Cohort	Bipolar disorder	DION1988 (Poor quality)	Hospitalisation	At admission, 34/44 people were unable to work; at 6 months post-release, 13/44 were unable to work (OR of unemployment at post-release compared with pre-release: 0.12, 95% CI 0.05, 0.32, *p* < 0.0001) At admission, 2/44 were employed at the expected level; at 6 months 9/44 were employed at the expected level (OR of employment 5.50 95% CI 1.09, 26.65, *p* = 0.038)
				TOHEN1990 (Fair quality)	Hospitalisation	After release from hospital, employment rates increased: 6 months post-hospital: 60% able to work or study; 48 months post-hospital: 72% able to work or study (significant improvement: McNemar X2 test, *p* = 0.002)
			Psychosis	TSIACHRISTOS2016 (Poor quality)	Early intervention in psychosis	People in the early intervention in psychosis group who were unemployed at baseline had an increased probability ratio, compared with people treated in other mental health services, of becoming employed (prevalence ratio [PR]: 2.16, 95% CI 1.26, 3.71, *p* = 0.005) at 3-year follow-up
		RCT	Bipolar disorder	PERRY1999 (low risk of bias)	Teaching patients with bipolar disorder to identify early symptoms of relapse and seek prompt treatment from health services	People given the intervention had improved employment rates compared with the control group at 18 months (mean difference 0.70, 95% CI 0.07, 1.33, measure range: 1–3). Improvements were not significantly different at 6 or 12 months
	Education attainment	Cohort	Psychosis	TSIACHRISTOS2016 (Poor quality)	Early intervention in psychosis	People in the early intervention in psychosis group did not show an increased probability of resuming studying compared with people treated in other mental health services (PR: 1.82, 95% CI 0.79, 4.21, *p* = 0.156)

*All results are in adult populations, and report intragenerational outcomes

#### Pharmacological interventions

##### Occupational status

*Paliperidone extended-release* One pre-post cohort study of poor quality [48] found that 52 weeks after adults with schizophrenia received treatment with Paliperidone extended release, the percentage of people in full-time competitive employment increased from pre-treatment (*p* < 0.0001).

*Olanzapine* One pre-post cohort study of poor quality [50] found that people with bipolar disorder treated with olanzapine for a mean of 28 weeks had reduced rates of paid employment (59.81% pre-treatment, 30.97% post-treatment, OR = 0.30, CI 0.17–0.53).

##### Summary: pharmacological interventions

Overall, evidence on the effectiveness of drug treatments in improving social mobility outcomes in people with SMI is extremely limited, mixed and of poor quality.

#### Psychosocial and service level interventions

##### Occupational status

*RCT evidence*

*Teaching patients with bipolar self-management* One RCT with a low ROB [49] found that people with bipolar disorder who were taught self-management techniques improved in terms of how well they performed in their employment (measured on a scale of 0–3) compared to the control group at 18 months (mean difference = 0.70, 95% CI 0.07–1.33). However, improvements at earlier time points (6 months, 12 months) were not significant.

*Non-RCT evidence*

*Early intervention in psychosis* One study of poor quality [12] found that people with psychosis treated with early intervention in psychosis services (low client-to-care-coordinator ratio, assertive community treatment, regular client appointments, routine psychological and family therapy for 3 years) who were unemployed at baseline had an increased probability ratio of becoming employed at 3-year follow-up compared to people who had standard care (2.16, CI 1.26–3.71, *p*  0.005).

*Hospitalisation* One pre-post study of fair quality [46] found that people with bipolar disorder admitted to hospital and treated at the discretion of the treating psychiatrist had significant improvements in employment rates at 48 months post-release compared to 6 months post-release (60% 6 months, 72% 48 months, McNemar *X*^2^ test: *p* = 0.002). Another study of poor quality [47] found that hospital admission reduced the odds of people with bipolar disorder being unemployed 6 months post-release, compared to before admission (OR = 0.12, CI 0.05–0.32, *p* < 0.0001). However, both these studies cannot control for any improvement in symptoms which was not the result of the treatment.

##### Educational attainment

*Non-RCT evidence* A study of poor quality [12] found that people treated in early intervention in psychosis services did not show an increased probability of resuming studying compared to people treated in other mental health services (1.82, CI 0.79–4.21, *p* = 0.156).

##### Summary: psychological and service level interventions

Limited, high quality RCT evidence suggests that teaching people with bipolar disorder to identify when to seek treatment for a relapse is beneficial in helping them maintain/gain employment. Fair quality observational evidence suggests that people with bipolar disorder also respond well to hospital admission with treatment according to psychiatrist discretion. Evidence for psychosis was of much poorer quality and extremely limited.

#### Summary: the association between interventions for mental health and social mobility outcomes

Overall, the evidence for associations between mental health treatment and improved social mobility is limited, particularly for pharmacological treatments. Given the large pool of research into pharmacological treatments, more work could be done to encourage the use of social mobility outcomes in clinical trials to better understand, how such interventions could improve social circumstances. Psychosocial interventions may provide some benefit in enabling people to return to or gain employment, for example, people with bipolar disorder. Evidence for improvements in other populations was limited.

## Discussion

This review found that social class is associated with interventions for mental health disorders, and that this link is multidimensional and reciprocal. Some evidence was found that social class is associated with access to treatment for people with mental health disorders. People from lower social classes appear to access treatment at later points on the care pathway, encountering crisis level intervention such as inpatient admission more often than people from higher social classes, who show more common contacts with primary and secondary care providers such as GPs and counselling services. This suggests that efforts should be made to increase accessibility to primary care for people from lower social classes, for example through additional funding in socially deprived areas [51] and more assertive outreach models [52].

Furthermore, people from lower social classes may not benefit from all interventions for mental health. More specific interventions, such as assigning a care manager, offering psychotherapy in place of pharmacotherapy, or considering educational level in deciding on treatment plan length may be required. Tailoring interventions may help to reduce the disparity in outcomes associated with low social class [53], a positive addition to work suggesting that adequate funding and improvement in quality of services in socially deprived areas can also ameliorate the effects of deprivation [51]. This may be particularly important given that low-income participants more commonly drop out of treatment than participants from higher-income backgrounds [14, 53], as this may reflect a feeling that current services are insufficient to address their needs.

Evidence for the impact of social class on treatment outcomes in people with SMI was notably lacking. It is important to understand how social class may impact treatment gains in this population, as not only can SMI be particularly distressing [54], but people tend to reach the point of intervention after a long period of experiencing symptoms [55], meaning that SMIs can have a particularly strong influence on downward social mobility [56]. A clearer understanding of the best ways to mitigate the negative influence of social class on intervention effectiveness would allow services to address this in intervention design, enabling a higher proportion of patients stuck in a cycle of crisis care and relapse to gain benefit. Moreover, there was some evidence that psychosocial intervention can help patients, particularly those with bipolar disorder, to return to employment which may provide optimism to patients. Possible adaptations to interventions should be evaluated in both controlled trials and longitudinal designs that evaluate long-term outcomes (including employment) of these interventions.

Given the vast body of literature on efficacy of pharmacological interventions [57, 58], surprisingly few reported social mobility outcomes. A fuller understanding of how treatment may impact experience of symptoms and also social mobility may help patients and clinicians make more informed treatment decisions.

### Limitations

There are some limitations associated with this review. Reporting of participant characteristics and methods was poor in some studies, with notable details often omitted from study descriptions. This may mean that important moderating variables of intervention effect were overlooked in our synthesis. Furthermore, the body of evidence addressing some of our research questions was limited, which meant that somewhat dated studies had more weight than they would have otherwise, notably data on the effects of inpatient admission on employment outcomes for people with SMIs [46, 47]. Similarly, some studies contributing to evidence on the impact of social class or socioeconomic status on treatment effectiveness were also relatively old [38, 43, 46]. It is important to consider the results of these studies within the context of their publication date, as the nature and perception of social class has changed over time [59], and therefore participants considered to be in one social class may not be considered to be in this social class in more recent studies. With this in mind, future work should seek to update the data regarding the impact of social class on treatment effectiveness as well as the impact of treatment on changes in social class, particularly in people with SMI.

Moreover, while employment was a focus in all six intervention studies found reporting social mobility outcomes, only one considered also return to education [12]. The impact of intervention on socio-economic status more generally, or income was also markedly lacking. Since social class can be conceived across a number of different domains [5], it is important to gain a full understanding of how treating mental health disorders can ameliorate the impact of symptoms on social mobility.

It is also important to note that included studies (with the exception of Kozma and colleagues, [48], who included participants from South Africa as well as the USA) are of populations which are predominantly White and of western origin. Findings are therefore limited in their generalisability to non-western nations who may have very different social classes, or limited differentiation of social class. Similarly, alongside the focus on predominantly Caucasian nations, 15 studies did not report the ethnicity of their sample, and only four studies reported ethnicity in detail. It is therefore not possible to explore within this review how ethnicity may interact with socio-economic status and mental health interventions.

Finally, researchers may have included social mobility measures in their studies but not reported them. A clearer protocol for examining the relationship between social class and mental health treatment and for reporting may aid future reviews in pooling a wider sample of relevant literature.

## Conclusion

In conclusion, social class is associated with the effectiveness of psychological interventions, but it may also be improved following treatment. Social class should be an inherent consideration in intervention design, both to prevent barriers to access and also to improve intervention effectiveness. Interventions should be adapted to allow benefits to be gained across social classes, and to ensure that robust measurement of social mobility outcomes is part of intervention trials. In turn, this may prevent social class from inhibiting potential treatment gains, weakening the association between poor mental health and reduced social mobility.

## Supplementary Information

Below is the link to the electronic supplementary material.

Supplementary file1 (DOCX 36 KB)

Supplementary file2 (DOCX 73 KB)

## References

[1] Isohanni I, Jones PB, Jarvelin MR, Nieminen P, Rantakallio P, Jokelainen J (2001). Educational consequences of mental disorders treated in hospital. A 31-year follow-up of the Northern Finland 1966 Birth Cohort. Psychol Med.

[2] Johnston DW, Schurer S, Shields MA (2013). Exploring the intergenerational persistence of mental health: evidence from three generations. J Health Econ.

[3] Sellers R, Warne N, Pickles A, Maughan B, Thapar A, Collishaw S (2019). Cross-cohort change in adolescent outcomes for children with mental health problems. J Child Psychol Psychiatry.

[4] Slominski L, Sameroff A, Rosenblum K, Kasser T (2011). Longitudinal predictors of adult socioeconomic attainment: the roles of socioeconomic status, academic competence, and mental health. Dev Psychopathol.

[5] Diemer MA, Mistry RS, Wadsworth ME, López I, Reimers F (2013). Best practices in conceptualizing and measuring social class in psychological research. Anal Soc Issues Public Policy.

[6] Bromberger JT, Schott LL, Matthews KA, Kravitz HM, Harlow SD, Montez JK (2017). Childhood socioeconomic circumstances and depressive symptom burden across 15 years of follow-up during midlife: Study of Women’s Health Across the Nation (SWAN). Arch Womens Ment Health.

[7] Silva M, Loureiro A, Cardoso G (2016). Social determinants of mental health: a review of the evidence. Eur J Psychiatry.

[8] Bor W, Dean AJ, Najman J, Hayatbakhsh R (2014). Are child and adolescent mental health problems increasing in the 21st century? A systematic review. Aust N Z J Psychiatry.

[9] Whiteford HA, Degenhardt L, Rehm J, Baxter AJ, Ferrari AJ, Erskine HE (2013). Global burden of disease attributable to mental and substance use disorders: findings from the Global Burden of Disease Study 2010. Lancet.

[10] McHugh RK, Barlow DH (2010). The dissemination and implementation of evidence-based psychological treatments: a review of current efforts. Am Psychol.

[11] Kodal A, Fjermestad KW, Bjelland I, Gjestad R, Ost LG, Bjaastad JF (2018). Predictors of long-term outcome of CBT for youth with anxiety disorders treated in community clinics. J Anxiety Disord.

[12] Tsiachristas A, Thomas T, Leal J, Lennox BR (2016). Economic impact of early intervention in psychosis services: results from a longitudinal retrospective controlled study in England. BMJ Open.

[13] Finegan M, Firth N, Wojnarowski C, Delgadillo J (2018). Associations between socioeconomic status and psychological therapy outcomes: a systematic review and meta-analysis. Depress Anxiety.

[14] Thompson MN, Cole OD, Nitzarim RS (2012). Recognizing social class in the psychotherapy relationship: a grounded theory exploration of low-income clients. J Couns Psychol.

[15] Moher D, Liberati A, Tetzlaff J, Altman DG (2010). Preferred reporting items for systematic reviews and meta-analyses: the PRISMA statement. PLoS Med.

[16] Centre for Reviews and Dissemination (2009) Systematic reviews: CRD's guidance for undertaking reviews in health care. Centre for Reviews and Dissemination, University of York

[17] Cooper C, Booth A, Varley-Campbell J, Britten N, Garside R (2018). Defining the process to literature searching in systematic reviews: a literature review of guidance and supporting studies. BMC Med Res Methodol.

[18] Cadth (2018) CADTH: Resources. https://www.cadth.ca/resources. Accessed 26 Nov 2021

[19] Scottish Intercollegiate Guidelines Network. Search filters [Internet]. https://www.sign.ac.uk/what-we-do/methodology/search-filters/. Accessed 26 Nov 2021

[20] Cooper C, Booth A, Britten N, Garside R (2017). A comparison of results of empirical studies of supplementary search techniques and recommendations in review methodology handbooks: a methodological review. Syst Rev.

[21] Briscoe S (2015). Web searching for systematic reviews: a case study of reporting standards in the UK health technology assessment programme. BMC Res Notes.

[22] Ouzzani M, Hammady H, Fedorowicz Z, Elmagarmid A (2016). Rayyan—a web and mobile app for systematic reviews. Syst Rev.

[23] Gondek D, Ning K, Ploubidis GB, Nasim B, Goodman A (2018). The impact of health on economic and social outcomes in the United Kingdom: a scoping literature review. PLoS ONE.

[24] Higgins JP, Altman DG, Gøtzsche PC, Jüni P, Moher D, Oxman AD (2011). The Cochrane Collaboration’s tool for assessing risk of bias in randomised trials. BMJ.

[25] Popay J, Roberts H, Sowden A, Petticrew M, Arai L, Rodgers M (2006). Guidance on the conduct of narrative synthesis in systematic reviews. Prod ESRC Methods Progr Version.

[26] Cummings JR (2014). Contextual socioeconomic status and mental health counseling use among US adolescents with depression. J Youth Adolesc.

[27] Dorner TE, Mittendorfer-Rutz E (2017). Socioeconomic inequalities in treatment of individuals with common mental disorders regarding subsequent development of mental illness. Soc Psychiatry Psychiatr Epidemiol.

[28] Paananen R, Santalahti P, Merikukka M, Rämö A, Wahlbeck K, Gissler M (2013). Socioeconomic and regional aspects in the use of specialized psychiatric care a Finnish nationwide follow-up study. Eur J Pub Health.

[29] Packness A, Waldorff FB, Christensen Rd, Hastrup LH, Simonsen E, Vestergaard M (2017). Impact of socioeconomic position and distance on mental health care utilization: a nationwide Danish follow-up study. Soc Psychiatry Psychiatr Epidemiol.

[30] Shah R, McNiece R, Majeed A (2001). General practice consultation rates for psychiatric disorders in patients aged 65 and over: prospective cohort study. Int J Geriatr Psychiatry.

[31] Bennett D, Rosenheck R (2020). Socioeconomic status and the effectiveness of treatment for first-episode psychosis. Health Serv Res.

[32] Button KS, Wiles NJ, Lewis G, Peters TJ, Kessler D (2012). Factors associated with differential response to online cognitive behavioural therapy. Soc Psychiatry Psychiatr Epidemiol.

[33] Cort NA, Gamble SA, Smith PN, Chaudron LH, Lu N, He H (2012). Predictors of treatment outcomes among depressed women with childhood sexual abuse histories. Depress Anxiety.

[34] Delgadillo J, Dawson A, Gilbody S, Bohnke JR (2017). Impact of long-term medical conditions on the outcomes of psychological therapy for depression and anxiety. Br J Psychiatry.

[35] El Alaoui S, Ljotsson B, Hedman E, Kaldo V, Andersson E, Ruck C (2015). Predictors of symptomatic change and adherence in internet-based cognitive behaviour therapy for social anxiety disorder in routine psychiatric care. PLoS ONE.

[36] Falconnier L (2009). Socioeconomic status in the treatment of depression. Am J Orthopsychiatry.

[37] Fournier JC, DeRubeis RJ, Shelton RC, Hollon SD, Amsterdam JD, Gallop R (2009). Prediction of response to medication and cognitive therapy in the treatment of moderate to severe depression. J Consult Clin Psychol.

[38] Gift TE, Strauss JS, Ritzler BA (1986). Social class and psychiatric outcome. Am J Psychiatry.

[39] Gilman SE, Fitzmaurice GM, Bruce ML, Have TT, Glymour MM, Carliner H (2013). Economic inequalities in the effectiveness of a primary care intervention for depression and suicidal ideation. Epidemiology.

[40] Hoyer J, Wiltink J, Hiller W, Miller R, Salzer S, Sarnowsky S (2016). Baseline patient characteristics predicting outcome and attrition in cognitive therapy for social phobia: results from a large multicentre trial. Clin Psychol Psychother.

[41] Joutsenniemi K, Laaksonen MA, Knekt P, Haaramo P, Lindfors O (2012). Prediction of the outcome of short- and long-term psychotherapy based on socio-demographic factors. J Affect Disord.

[42] Kelly JM, Jakubovski E, Bloch MH (2015). Prognostic subgroups for remission and response in the coordinated anxiety learning and management (CALM) trial. J Clin Psychiatry.

[43] Myers JK, Bean LL, Pepper MP (1965). Social class and psychiatric disorders: a ten year follow-up. J Health Hum Behav.

[44] Pirkis J, Bassilios B, Fletcher J, Sanderson K, Spittal MJ, King K (2011). Clinical improvement after treatment provided through the Better Outcomes in Mental Health Care (BOiMHC) programme: do some patients show greater improvement than others?. Aust N Z J Psychiatry.

[45] Poots AJ, Green SA, Honeybourne E, Green J, Woodcock T, Barnes R (2014). Improving mental health outcomes: achieving equity through quality improvement. Int J Qual Health Care.

[46] Tohen M, Waternaux CM, Tsuang MT (1990). Outcome in mania: a 4-year prospective follow-up of 75 patients utilizing survival analysis. Arch Gen Psychiatry.

[47] Dion GL, Tohen M, Anthony WA, Waternaux CS (1988). Symptoms and functioning of patients with bipolar disorder six months after hospitalization. Psychiatr Serv.

[48] Kozma C, Dirani R, Canuso C, Mao L (2011). Change in employment status over 52 weeks in patients with schizophrenia: an observational study. Curr Med Res Opin.

[49] Perry A, Tarrier N, Morriss R, McCarthy E, Limb K (1999). Randomised controlled trial of efficacy of teaching patients with bipolar disorder to identify early symptoms of relapse and obtain treatment. BMJ.

[50] Roy Chengappa K, Hennen J, Baldessarini RJ, Kupfer DJ, Yatham LN, Gershon S (2005). Recovery and functional outcomes following olanzapine treatment for bipolar I mania. Bipolar Disord.

[51] Clark DM, Canvin L, Green J, Layard R, Pilling S, Janecka MJ (2018). Transparency about the outcomes of mental health services (IAPT approach): an analysis of public data. Lancet.

[52] Delgadillo J, Farnfield A, North A (2018). Social inequalities in the demand, supply and utilisation of psychological treatment. Couns Psychother Res.

[53] Goodman LA, Pugach M, Skolnik A, Smith L (2013). Poverty and mental health practice: within and beyond the 50-minute hour. J Clin Psychol.

[54] Saunders JC (2003). Families living with severe mental illness: a literature review. Issues Ment Health Nurs.

[55] McGorry PD, Killackey E, Yung AR (2007). Early intervention in psychotic disorders: detection and treatment of the first episode and the critical early stages. Med J Aust.

[56] O'Donoghue B, Lyne JP, Fanning F, Kinsella A, Lane A, Turner N (2014). Social class mobility in first episode psychosis and the association with depression, hopelessness and suicidality. Schizophr Res.

[57] Mayo-Wilson E, Dias S, Mavranezouli I, Kew K, Clark DM, Ades A (2014). Psychological and pharmacological interventions for social anxiety disorder in adults: a systematic review and network meta-analysis. The Lancet Psychiatry.

[58] Murphy BP, Chung Y-C, Park T-W, McGorry PD (2006). Pharmacological treatment of primary negative symptoms in schizophrenia: a systematic review. Schizophr Res.

[59] Cohen D, Shin F, Liu X, Ondish P, Kraus MW (2017). Defining social class across time and between groups. Pers Soc Psychol Bull.

