# Supplementary material for: The association between social class and the impact of treatment for mental health problems: a systematic review and narrative synthesis
Source: Soc Psychiatry Psychiatr Epidemiol. 2022 Nov 23;58(4):581–603. doi: 10.1007/s00127-022-02378-9 (PMC10066076; doi:10.1007/s00127-022-02378-9)
Supplement: Supplementary file 1 — Supplementary file1 (DOCX 36 KB) [file 127_2022_2378_MOESM1_ESM.docx]

*The Association between Social Class and the Impact of Treatment for Mental Health Problems: A Systematic Review and Narrative Synthesis.*

*Social Psychiatry and Psychiatric Epidemiology*

*Phoebe Barnett, Iyinoluwa Oshinowo, Christopher Cooper, Clare Taylor, Shubulade Smith, Stephen Pilling*

*Corresponding author:*

*Phoebe Barnett*

[*phoebe.barnett@ucl.ac.uk*](mailto:phoebe.barnett@ucl.ac.uk)

*Centre for Outcomes Research and Effectiveness, Research Department of Clinical, Educational and Health Psychology, University College London, London, UK*

**Online resource 1: Protocol**

## Research question

**Does social class moderate the impact of treatments for mental disorders?**

2.1 What is the effect of social class on the likelihood of receiving treatment for mental disorders?

2.2 What is the effect of social class on treatment outcome for mental disorders?

2.3 What is the effect of treatment versus no treatment for mental disorders on influencing class change?

## Methods

The review will be carried out according to the methodological guidance set out in the Centre for Reviews and Dissemination’s handbook (1).

### Searching

Study identification (literature searching) will use database and non-database methods of study identification (2).

Bibliographic

The following electronic databases will be searched:

- Ovid MEDLINE(R) and Epub Ahead of Print, In-Process & Other Non-Indexed Citations and Daily*
- Embase 1974 to 2019 September 24*
- HMIC Health Management Information Consortium 1979 to May 2019*
- PsycINFO 1806 to September Week 2 2019*
- Social Policy and Practice 201907*
- Applied Social Sciences Index & Abstracts (ASSIA)**
- Social Science Database**
- ERIC***

* searched via Ovid; ** searched via ProQuest; *** searched via EBSCOHost.

The bibliographic search strategy will take the following form:

((search terms for social mobility) AND (search terms for mental health) and (studies reporting RCT or Cohort/longitudinal study designs))

The following study deign literature search filters will be used: the CADATH RCT/CCT filter (3) and the SIGN filter (4). The SIGN filter was adapted to focus on studies reporting cohort or longitudinal designs.

The search strategy will be reviewed using the PRESS Checklist (5). The primary search strategy is reported in appendix two using a search narrative to explain the contextual and conceptual detail behind the selection of search terms (6).

The following non-database search methods will be undertaken to identify published and unpublished studies:

- Relevamt Systematic reviews will be checked and their included studies cross-checked for any studies that might meet our inclusion criteria but which have not been identified by our bibliographic searches;
- Web-searching will be undertaken by one researcher using the Google advanced interface. The search strategies will be based on the bibliographic search strategy. The guidance of Briscoe will be used to report web-searches (7);

*Limits*

The searches will not be limited by date or language of publication. The searches will be limited by study design to: studies reporting trials (e.g. RCTs) or cohort studies. Systematic reviews will not be included but any identified will be retained for scrutiny of their included studies (see above).

## Screening

All studies will be double screened at title/abstract and full-text by three researches using Rayyan software (8). The title/abstract screening is initially broader in scope than is necessary to address this research question specifically. The searches and title/abstract screening are being used to address a second research question and separate review.

The following inclusion criteria will be applied at title/abstract:

Study reports:

1. An RCT OR Cohort study and
2. Population has a mental disorder; and
3. Social mobility is an outcome of the study.

Criteria 2 and 3 will be interpreted broadly with any recognised mental disorder or possible description of social mobility being sufficient for a study to be included for further screening at full-text.

The following inclusion criteria will be applied at full-text:

Study reports:

1. RCT OR Cohort OR Systematic review; and
2. Population has a mental disorder; and
3. Social mobility is an outcome of the study; and
4. Intervention/treatment for mental disorder is described with reference to results

Criteria 1 and 4 are intended to focus study selection at full-text to align with the research question of this review specifically. The decision to limit studies reporting RCT or cohort studies was taken by the expert reference group.

## Quality appraisal

Quality appraisal will be undertaken by one reviewer and checked in detail by another. The Cochrane Risk of Bias tool will be used for studies reporting RCT (9) and the Newcastle-Ottawa tool will be used for Cohort studies (10).

## Data extraction

The following data will be extracted by one reviewer and checked in detail by another.

For RCT

- Bib ref
- Study type
- Study quality
- Intervention
- Comparator
- Method of allocation
- Setting
- N of participants
- Participant characteristics
- Length of follow-up
- Method of analysis
- Outcomes
- Unintended consequences
- Limitations identified by authors
- Funding

For cohort studies

Descriptive data

Study ref

- Selection/eligibility criteria for cohort
- Cohort/sample size
- Age: mean sample age
- Gender: % of sample of who are female
- Ethnicity: % of each ethnicity recorded
- Diagnosis (inc. diagnostic criteria used)
- Specific disorder + formal diagnosis (y/n) and criteria used

Outcome data for synthesis

- Comparator or intervention/ exposure
- Occupational change (method of measurement and assessment will be extracted)
- Change in income or socioeconomic status (method of measurement and assessment will be extracted)
- Severity of illness
- Events or sum. measures over-time
- Adjusted = if yes, how
- Data collection points and follow-up
- Potential confounders/modifiers
- Ref to missing data or loss to follow-up
- Country
- Region
- Setting

General data

- Limitations as identified by study authors
- Funding
- Link to other work (protocol or other studies)

## Data synthesis

Data will be synthesised narratively by mental disorder (e.g., anxiety or depressive disorder) and then sub-categorised by identified social mobility outcomes. Similar outcomes will be grouped and differences between study outcomes explained narratively. Study characteristics will be reported in three tables aligned to the subheadings set out above in data-extraction.

If sufficiently homogenous and high-quality data are located, random-effects meta-analysis may be considered, although this is unlikely. A minimum number of five studies reporting unadjusted data and the same social mobility outcome measures will be used as initial guide.

## Review team

Phoebe Barnett

Chris Cooper

Iyinoluwa Oshinowo

Stephen Pilling

## References

1. Centre for Reviews and Dissemination. Systematic reviews – CRD’s guidance for undertaking reviews in healthcare. York: Centre for Reviews and Dissemination, University of York; 2009. Available from: https://www.york.ac.uk/media/crd/Systematic_Reviews.pdf.

2. Cooper C, Booth A, Varley-Campbell J, Britten N, Garside R. Defining the process to literature searching in systematic reviews: a literature review of guidance and supporting studies. BMC Medical Research Methodology. 2018;18(1):85.

3. Cadth. CADTH: Resources 2018 [Available from: https://www.cadth.ca/resources.

4. Network SIG. SIGN Strategy (Ovid) [Available from: http://www.sign.ac.uk/assets/search-filters-observational-studies.docx.

5. McGowan J, Sampson M, Salzwedel DM, Cogo E, Foerster V, Lefebvre C. PRESS Peer Review of Electronic Search Strategies: 2015 Guideline Statement. Journal of clinical epidemiology. 2016;75:40-6.

6. Cooper C, Dawson S, Peters J, Varley-Campbell J, Cockcroft E, Hendon J, et al. Revisiting the need for a literature search narrative: A brief methodological note. Research synthesis methods. 2018.

7. Briscoe S. Web searching for systematic reviews: a case study of reporting standards in the UK Health Technology Assessment programme. BMC research notes. 2015;8:153.

8. Mourad O, Hossam H, Zbys F, Elmagarmid A. Rayyan — a web and mobile app for systematic reviews. Systematic Reviews. 2016;5(210).

9. Higgins JPT, Altman DG, Gøtzsche PC, Jüni P, Moher D, Oxman AD, et al. The Cochrane Collaboration’s tool for assessing risk of bias in randomised trials. BMJ. 2011;343:d5928.

10. GA Wells BS, D O'Connell, J Peterson, V Welch, M Losos, P Tugwell,,. The Newcastle-Ottawa Scale (NOS) for assessing the quality of nonrandomised studies in meta-analyses, 2019 [Available from: http://www.ohri.ca/programs/clinical_epidemiology/oxford.asp.

11. Lefebvre C, Manheimer E, Glanville J. Chapter 6: Searching for studies. 2011 [cited Accessed 7th December 2017]. In: Cochrane Handbook for Systematic Reviews of Interventions [Internet]. The Cochrane Collaboration, [cited Accessed 7th December 2017]. Available from: http://handbook.cochrane.org/.

12. DRAFT REPORT ON FOSTERING SOCIAL MOBILITY AS A CONTRIBUTION TO SOCIAL COHESION,. Strasbourg: EUROPEAN COMMITTEE OF SOCIAL COHESION (CDCS),; 2011.

13. Social mobility indicators Gov.Uk: The Deputy Prime Minister’s Office; 2015 [Available from: https://www.gov.uk/government/publications/social-mobility-indicators/social-mobility-indicators.

14. Department for business invovation and skils. Social Mobility: A literature review. London: Department for business invovation and skils,; 2011.

15. Elhakeem A, Hardy R, Bann D, Caleyachetty R, Cosco TD, Hayhoe RP, et al. Intergenerational social mobility and leisure-time physical activity in adulthood: a systematic review. Journal of Epidemiology and Community Health. 2017;71(7):673-80.

16. Group BC. THE STATE OF SOCIAL MOBILITY IN THE UK. UK: Sutton Trust; 2017.

17. Motta JVdS, Lima NP, Olinto MTA, Gigante DP. Social mobility and smoking: a systematic review. Ciência & Saúde Coletiva. 2015;20:1515-20.

18. Torche F. How do we characteristically measure and analyze intergenerational mobility? 2013.

19. de Quadros Lde C, Laura HC, Quevedo Lde A, Gigante DP. [Effects of social mobility on adult mental health:a systematic review of the literature]. Cien Saude Colet. 2016;21(2):443-8.

20. Finegan M, Firth N, Wojnarowski C, Delgadillo J. Associations between socioeconomic status and psychological therapy outcomes: A systematic review and meta-analysis. Depression and anxiety. 2018;35(6):560-73.

21. Hoven H, Siegrist J, Goldberg M, Ribet C, Zins M, Wahrendorf M. Intragenerational social mobility and depressive symptoms. Results from the French CONSTANCES cohort study. SSM - Population Health. 2019;7:100351.

22. The Canadian Agency for Drugs and Technologies in Health (CADTH). Strings Attached: CADTH’s Database Search FIlters 2018 [Available from: https://www.cadth.ca/resources/finding-evidence/strings-attached-cadths-database-search-filters#guide.

23. Cooper C, Varley-Campbell J, Carter P. Established search filters may miss studies when identifying randomised controlled trials. Journal of Clinical Epidemiology. 2019;112(August):12-9.

24. Healthcare Improvement Scotland: SIGN. Search Filters Edinburgh: Healthcare Improvement Scotland: SIGN; 2019 [cited 2019 Jul 19]. Available from: https://www.sign.ac.uk/search-filters.html.
