# Supplementary material for: The association between social class and the impact of treatment for mental health problems: a systematic review and narrative synthesis
Source: Soc Psychiatry Psychiatr Epidemiol. 2022 Nov 23;58(4):581–603. doi: 10.1007/s00127-022-02378-9 (PMC10066076; doi:10.1007/s00127-022-02378-9)
Supplement: Supplementary file 2 — Supplementary file2 (DOCX 73 KB) [file 127_2022_2378_MOESM2_ESM.docx]

*The Association between Social Class and the Impact of Treatment for Mental Health Problems: A Systematic Review and Narrative Synthesis.*

*Social Psychiatry and Psychiatric Epidemiology*

*Phoebe Barnett, Iyinoluwa Oshinowo, Christopher Cooper, Clare Taylor, Shubulade Smith, Stephen Pilling*

*Corresponding author:*

*Phoebe Barnett*

[*phoebe.barnett@ucl.ac.uk*](mailto:phoebe.barnett@ucl.ac.uk)

*Centre for Outcomes Research and Effectiveness, Research Department of Clinical, Educational and Health Psychology, University College London, London, UK*

Online resource 2: Full search strategy

**UPDATE SEARCH CONDUCTED 17th March 2021**

**MEDLINE UPDATE: Before dupes = 186 additional**

**EMBASE update: 229 additional**

**PsychINFO update 123 additional**

**Social policy and practice update 5 additional**

**HMIC update 1 additional**

**ASSIA update 14 additional**

**ERIC update 0 additional**

**Combined= 558**

**Deduped= 476**

**Included ti/ab=13**

**Included FT=1**

**Excluded: 12**

**Database:** Ovid MEDLINE(R) and Epub Ahead of Print, In-Process & Other Non-Indexed Citations and Daily <1946 to September

27, 2019>

**Host:** OVID

**Search parameters:** 1946 to September 27, 2019

**Date of search:** Monday September 30^th^ 2019

**Search checked by:** Phoebe Barnett and Iyinoluwa Oshinowo.

**Conceptual narrative:** The purpose of this literature search is to identify studies which examine the impact of mental health conditions on social mobility. The units of analysis will be: i) studies reporting randomised controlled trials (RCT), to examine access to treatment (or interventions) by social class, and; ii) cohort studies, to examine the impact of mental health conditions on social mobility over time. The decision to focus on these particular study designs was undertaken by the project working group which includes world-leaders in the field.

Below, a contextual narrative is set out to describe the structure and technical detail of the literature search, as well as to explain the decision-making process of the literature search for MEDLINE (1).

| Search strategy | Contextual narrative |
| --- | --- |
| 1     Social Mobility/ (959)  2     (Social$ adj3 (mobil$ or change)).ti,ab,kw. (7073)  3     ((absolute or relative or "long range" or "short range") adj3 mobil$).ti,ab,kw. (1785)  4     exp Socioeconomic Factors/ and mobil$.ti,ab,kw. (5716)  5     *Economic Status/ and (mobil$ or change).ti,ab,kw. (6)  6     ((socioeconomic or socio-economic or economic$ or income or salary or salaries or earn or earning or wealth or  occupation or employment or job) adj3 (mobil$ or change)).ti,ab,kw. (3107)  7     ((intergenerational or inter-generational or Intragenerational or Intra-generational) adj2 (mobil$ or  change)).ti,ab,kw. (208)  8     ((low or medium or high) adj3 depriv$).ti,ab,kw. (1699)  9     ((father$ or mother$ or parent$ or grandfather$ or grandmother$ or early-life) adj3 (occupation* or employment$ or  job or education or upbringing)).ti,ab,kw. (15419)  10     *Social class/ (12823)  11     ((social class or social background) adj3 (mobil$ or change)).ti,ab,kw. (84)  12     ((poor or disadvantage$) adj3 (background or mobil$)).ti,ab,kw. (3119)  13     *Education/ and mobil$.ti,ab,kw. (138)  14     ((education$ and (attainment or performance or results or outcome$ or grade$)) adj3 mobil$).ti,ab,kw. (2106)  15     1 or 2 or 3 or 4 or 5 or 6 or 7 or 8 or 9 or 10 or 11 or 12 or 13 or 14 (49942) | Lines 1-14 set out the search terms for social mobility. The search terms are reported by line number (and as run, per Cochrane guidance (2)), with the corresponding number of studies identified per line reported in parentheses.  Lines 1, 4, 5 and 13 represent controlled indexing terminology (identified by the use of /).  Line 5, 10 and 13 represent focused indexing terms (identified by the *) which means only studies in which the indexing term is the central topic of the paper will be retrieved.  Lines 5 and 13 are further focused by free-text search terms, meaning that the search line searches first for the indexing term and then within this, any study using the term mobil* at title, abstract or keyword.  The remaining lines represent free-text search terms. Free-text terminology was identified in scoping, a review of the search terms used in potentially relevant systematic reviews and their included studies, and with reference to our project reference group (c.f. (3-12)). All the searching is undertaken on the title (ti), abstract (ab) and author generated keyword (kw) fields.  The free-text lines use proximity searching, indicated by adj3. This means that the term social* will be searched within two words of the term mobil*, allowing for other words which may appear between these two words, and in any order.  Truncation is used indicated by the $ sign. Truncation captures alternative spellings or word-endings, such as social$ for socially and mobil$ for mobile or mobility.  Line 15 combines all of the search terms using the Boolean connector OR. This means that all of the search terms set out in lines 1-14 will be searched for. |
| 16     exp Mental Disorders/ (1192680)  17     (mental$ adj3 (health$ or ill$ or unwell$ or disorder$)).ti,ab,kw. (189878)  18     16 or 17 (1287141) | Line 16 represents controlled indexing for mental disorders. The term is employed to capture relevant sub-headings (represented by exp). In this case, the sub-headings relate to mental health conditions such as anxiety or depression.  Line 17 represents high-level terms for mental health terminology. Given the unit of analysis for this reviews, and the resources available, this is a pragmatic decision to control the volume of studies identified (2). A form of sensitivity analysis has been undertaken to examine the effect of this decision and recall was unaffected based on the marker papers identified from scoping searches. |
| 19     (Randomized Controlled Trial or Controlled Clinical Trial or Pragmatic Clinical Trial or Equivalence Trial or  Clinical Trial, Phase III).pt. (581627)  20     Randomized Controlled Trial/ (490235)  21     exp Randomized Controlled Trials as Topic/ (129274)  22     "Randomized Controlled Trial (topic)"/ (0)  23     Controlled Clinical Trial/ (93274)  24     exp Controlled Clinical Trials as Topic/ (134385)  25     "Controlled Clinical Trial (topic)"/ (0)  26     Randomization/ (100536)  27     Random Allocation/ (100536)  28     Double-Blind Method/ (153495)  29     Double Blind Procedure/ (0)  30     Double-Blind Studies/ (153495)  31     Single-Blind Method/ (27365)  32     Single Blind Procedure/ (0)  33     Single-Blind Studies/ (27365)  34     Placebos/ (34468)  35     Placebo/ (0)  36     Control Group/ (1631)  37     (random* or sham or placebo*).ti,ab,hw,kf,kw. (1424589)  38     ((singl* or doubl*) adj (blind* or dumm* or mask*)).ti,ab,hw,kf,kw. (228120)  39     ((tripl* or trebl*) adj (blind* or dumm* or mask*)).ti,ab,hw,kf,kw. (908)  40     (control* adj3 (study or studies or trial* or group*)).ti,ab,kf,kw. (928554)  41     (Nonrandom* or non random* or non-random* or quasi-random* or quasirandom*).ti,ab,hw,kf,kw. (41466)  42     allocated.ti,ab,hw. (61744)  43     ((open label or open-label) adj5 (study or studies or trial*)).ti,ab,hw,kf,kw. (32418)  44     ((equivalence or superiority or non-inferiority or noninferiority) adj3 (study or studies or  trial*)).ti,ab,hw,kf,kw. (7380)  45     (pragmatic study or pragmatic studies).ti,ab,hw,kf,kw. (361)  46     ((pragmatic or practical) adj3 trial*).ti,ab,hw,kf,kw. (4389)  47     ((quasiexperimental or quasi-experimental) adj3 (study or studies or trial*)).ti,ab,hw,kf,kw. (6931)  48     (phase adj3 (III or "3") adj3 (study or studies or trial*)).ti,hw,kf,kw. (26242)  49     or/19-48 (2051645) | Lines 19-48 are search terms for controlled trials. The search filter used here is the Canadian Agency for Drugs and Technologies in Health (CADTH) April 2018 controlled trials filter (13). The filter has been amended at line 48 to incorporate the P3 search filter (14). The P3 filter improves the sensitivity of searches for controlled trials. |
| 50     exp cohort studies/ (1902507)  51     (cohort adj (study or studies)).tw. (184913)  52     Cohort analy$.tw. (7266)  53     (Follow up adj (study or studies)).tw. (47556)  54     (observational adj (study or studies)).tw. (96396)  55     Longitudinal.tw. (228668)  56     Retrospective.tw. (487730)  57     50 or 51 or 52 or 53 or 54 or 55 or 56 (2273502) | Lines 50-56 are search terms for cohort studies (and potential synonyms) which are taken from the Scottish Intercollegiate Guidelines Network (SIGN) observational search filter (15). |
| 58     49 or 57 (3958780) | Line 58 combines the search filter for controlled trials (line 49) OR the search filter for cohort studies (line 57). |
| 59     15 and 18 and 58 (2168) | Line 59 completes the literature search, combining line 15 (terms for social mobility) AND (terms for mental health conditions) AND (terms for controlled trials OR cohort studies)  No language or date limits are applied to the search strategy. |

**Database:** Embase

**Host:** OVID

**Search parameters:** 1980 to 2019 Week 39

**Date of search:** Monday September 30^th^ 2019

Search Strategy:

| **#** | **Searches** | **Results** |
| --- | --- | --- |
| 1 | social class/ | 28742 |
| 2 | (Social$ adj3 (mobil$ or change)).ti,ab,kw. | 7520 |
| 3 | ((absolute or relative or "long range" or "short range") adj3 mobil$).ti,ab,kw. | 1686 |
| 4 | exp socioeconomics/ and mobil$.ti,ab,kw. | 5267 |
| 5 | (Economic Status and (mobil$ or change)).ti,ab,kw. | 1227 |
| 6 | ((socioeconomic or socio-economic or economic$ or income or salary or salaries or earn or earning or wealth or occupation or employment or job) adj3 (mobil$ or change)).ti,ab,kw. | 3390 |
| 7 | ((intergenerational or inter-generational Intragenerational or Intra-generational) adj2 (mobil$ or change)).ti,ab,kw. | 185 |
| 8 | ((low or medium or high) adj3 depriv$).ti,ab,kw. | 2102 |
| 9 | ((father$ or mother$ or parent$ or grandfather$ or grandmother$ or early-life) adj3 (occupation* or employment$ or job or education or upbringing)).ti,ab,kw. | 18492 |
| 10 | ((social class or social background) adj3 (mobil$ or change)).ti,ab,kw. | 75 |
| 11 | ((poor or disadvantage$) adj3 (background or mobil$)).ti,ab,kw. | 4986 |
| 12 | *Education/ and mobil$.ti,ab,kw. | 693 |
| 13 | ((education$ and (attainment or performance or results or outcome$ or grade$)) adj3 mobil$).ti,ab,kw. | 3191 |
| 14 | 1 or 2 or 3 or 4 or 5 or 6 or 7 or 8 or 9 or 10 or 11 or 12 or 13 | 73031 |
| 15 | exp mental disease/ | 2002435 |
| 16 | (mental$ adj3 (health$ or ill$ or unwell$ or disorder$)).ti,ab,kw. | 241963 |
| 17 | 15 or 16 | 2084340 |
| 18 | (Randomized Controlled Trial or Controlled Clinical Trial or Pragmatic Clinical Trial or Equivalence Trial or Clinical Trial, Phase III).pt. | 0 |
| 19 | Randomized Controlled Trial/ | 569931 |
| 20 | exp Randomized Controlled Trials as Topic/ | 169586 |
| 21 | "Randomized Controlled Trial (topic)"/ | 169586 |
| 22 | Controlled Clinical Trial/ | 465567 |
| 23 | exp Controlled Clinical Trials as Topic/ | 176505 |
| 24 | "Controlled Clinical Trial (topic)"/ | 10424 |
| 25 | Randomization/ | 84358 |
| 26 | Random Allocation/ | 80621 |
| 27 | Double-Blind Method/ | 130266 |
| 28 | Double Blind Procedure/ | 163633 |
| 29 | Double-Blind Studies/ | 124188 |
| 30 | Single-Blind Method/ | 34831 |
| 31 | Single Blind Procedure/ | 36774 |
| 32 | Single-Blind Studies/ | 36774 |
| 33 | Placebos/ | 272486 |
| 34 | Placebo/ | 329404 |
| 35 | Control Group/ | 110796 |
| 36 | (random* or sham or placebo*).ti,ab,hw,kw. | 2141950 |
| 37 | ((singl* or doubl*) adj (blind* or dumm* or mask*)).ti,ab,hw,kw. | 311362 |
| 38 | ((tripl* or trebl*) adj (blind* or dumm* or mask*)).ti,ab,hw,kw. | 1491 |
| 39 | (control* adj3 (study or studies or trial* or group*)).ti,ab,kw. | 1439000 |
| 40 | (Nonrandom* or non random* or non-random* or quasi-random* or quasirandom*).ti,ab,hw,kw. | 58156 |
| 41 | allocated.ti,ab,hw. | 79527 |
| 42 | ((open label or open-label) adj5 (study or studies or trial*)).ti,ab,hw,kw. | 68574 |
| 43 | ((equivalence or superiority or non-inferiority or noninferiority) adj3 (study or studies or trial*)).ti,ab,hw,kw. | 13643 |
| 44 | (pragmatic study or pragmatic studies).ti,ab,hw,kw. | 671 |
| 45 | ((pragmatic or practical) adj3 trial*).ti,ab,hw,kw. | 6163 |
| 46 | ((quasiexperimental or quasi-experimental) adj3 (study or studies or trial*)).ti,ab,hw,kw. | 14045 |
| 47 | (phase adj3 (III or "3") adj3 (study or studies or trial*)).ti,hw,kw. | 98884 |
| 48 | or/18-47 | 1340348 |
| 49 | Longitudinal study/ | 130965 |
| 50 | Retrospective study/ | 830519 |
| 51 | Prospective study/ | 554136 |
| 52 | Cohort analysis/ | 513274 |
| 53 | (Cohort adj (study or studies)).mp. | 278623 |
| 54 | (follow up adj (study or studies)).tw. | 58300 |
| 55 | (observational adj (study or studies)).tw. | 152831 |
| 56 | 49 or 50 or 51 or 52 or 53 or 54 or 55 | 1934416 |
| 57 | (systematic adj3 review$).ti,ab,kw. | 203764 |
| 58 | 48 or 56 or 57 | 3235554 |
| 59 | 14 and 17 and 58 | 2776 |

**Database:** PsycINFO

**Host:** OVID

**Search parameters:** 1806 to September Week 2 2019

**Date of search:** Monday September 30^th^ 2019

| **#** | **Searches** | **Results** |
| --- | --- | --- |
| 1 | exp Clinical Trials/ or Placebo/ or (random* or sham or placebo* or ((singl* or doubl*) adj (blind* or dumm* or mask*)) or ((tripl* or trebl*) adj (blind* or dumm* or mask*)) or (control* adj3 (study or studies or trial* or group*)) or Nonrandom* or non random* or non-random* or quasi-random* or quasirandom* or allocated or ((open label or open-label) adj5 (study or studies or trial*)) or ((equivalence or superiority or non-inferiority or noninferiority) adj3 (study or studies or trial*)) or ((pragmatic or practical) adj3 trial*) or ((quasiexperimental or quasi-experimental) adj3 (study or studies or trial*)) or (phase adj3 (III or "3") adj3 (study or studies or trial*))).ti,ab,hw. | 360650 |
| 2 | Randomized Controlled Trial/ | 130 |
| 3 | Placebo/ | 5351 |
| 4 | Control Group/ | 910 |
| 5 | (random* or sham or placebo*).ti,ab,hw. | 221588 |
| 6 | ((singl* or doubl*) adj (blind* or dumm* or mask*)).ti,ab,hw. | 25262 |
| 7 | ((tripl* or trebl*) adj (blind* or dumm* or mask*)).ti,ab,hw. | 74 |
| 8 | (control* adj3 (study or studies or trial* or group*)).ti,ab. | 159024 |
| 9 | (Nonrandom* or non random* or non-random* or quasi-random* or quasirandom*).ti,ab,hw. | 4895 |
| 10 | allocated.ti,ab,hw. | 10043 |
| 11 | ((open label or open-label) adj5 (study or studies or trial*)).ti,ab,hw. | 4402 |
| 12 | ((equivalence or superiority or non-inferiority or noninferiority) adj3 (study or studies or trial*)).ti,ab,hw. | 861 |
| 13 | (pragmatic study or pragmatic studies).ti,ab,hw. | 95 |
| 14 | ((pragmatic or practical) adj3 trial*).ti,ab,hw. | 698 |
| 15 | ((quasiexperimental or quasi-experimental) adj3 (study or studies or trial*)).ti,ab,hw. | 4811 |
| 16 | (phase adj3 (III or "3") adj3 (study or studies or trial*)).ti,ab. | 1564 |
| 17 | Or/1-16 | 329220 |
| 18 | (cohort adj (study or studies)).tw. | 19931 |
| 19 | Cohort analy$.tw. | 858 |
| 20 | (Follow up adj (study or studies)).tw. | 12676 |
| 21 | (observational adj (study or studies)).tw. | 9693 |
| 22 | Longitudinal.tw. | 110858 |
| 23 | Retrospective.tw. | 33797 |
| 24 | Or/18-23 | 174997 |
| 25 | (systematic adj3 review$).ti,ab. | 29000 |
| 26 | 17 or 24 or 25 | 504680 |
| 27 | Social Mobility/ | 1562 |
| 28 | (Social$ adj3 (mobil$ or change)).ti,ab. | 20566 |
| 29 | ((absolute or relative or "long range" or "short range") adj3 mobil$).ti,ab. | 111 |
| 30 | exp Socioeconomic Status/ and mobil$.ti,ab. | 1659 |
| 31 | (Economic Status and (mobil$ or change)).ti,ab. | 585 |
| 32 | ((socioeconomic or socio-economic or economic$ or income or salary or salaries or earn or earning or wealth or occupation or employment or job) adj3 (mobil$ or change)).ti,ab. | 3222 |
| 33 | ((intergenerational or inter-generational Intragenerational or Intra-generational) adj2 (mobil$ or change)).ti,ab. | 284 |
| 34 | ((low or medium or high) adj3 depriv$).ti,ab. | 497 |
| 35 | ((father$ or mother$ or parent$ or grandfather$ or grandmother$ or early-life) adj3 (occupation* or employment$ or job or education or upbringing)).ti,ab. | 14589 |
| 36 | *Social Class/ | 5342 |
| 37 | ((social class or social background) adj3 (mobil$ or change)).ti,ab. | 118 |
| 38 | ((poor or disadvantage$) adj3 (background or mobil$)).ti,ab. | 640 |
| 39 | *Education/ and mobil$.ti,ab. | 395 |
| 40 | ((education$ and (attainment or performance or results or outcome$ or grade$)) adj3 mobil$).ti,ab. | 1241 |
| 41 | Or/27-40 | 46738 |
| 42 | exp Mental Disorders/ | 825868 |
| 43 | (mental$ adj3 (health$ or ill$ or unwell$ or disorder$)).ti,ab. | 241719 |
| 44 | 42 or 43 | 958106 |
| 45 | 26 and 41 and 44 | 1151 |

**Database:** Social Policy and Practice (SPP)

**Host:** OVID

**Search parameters:** 201907

**Date of search:** Monday September 30^th^ 2019

Search Strategy:

| **#** | **Searches** | **Results** |
| --- | --- | --- |
| 1 | (random* or sham or placebo*).ti,ab,hw. | 4936 |
| 2 | ((singl* or doubl*) adj (blind* or dumm* or mask*)).ti,ab,hw. | 89 |
| 3 | ((tripl* or trebl*) adj (blind* or dumm* or mask*)).ti,ab,hw. | 0 |
| 4 | (control* adj3 (study or studies or trial* or group*)).ti,ab. | 3626 |
| 5 | (Nonrandom* or non random* or non-random* or quasi-random* or quasirandom*).ti,ab,hw. | 162 |
| 6 | allocated.ti,ab,hw. | 861 |
| 7 | ((open label or open-label) adj5 (study or studies or trial*)).ti,ab,hw. | 8 |
| 8 | ((equivalence or superiority or non-inferiority or noninferiority) adj3 (study or studies or trial*)).ti,ab,hw. | 13 |
| 9 | (pragmatic study or pragmatic studies).ti,ab,hw. | 1 |
| 10 | ((pragmatic or practical) adj3 trial*).ti,ab,hw. | 39 |
| 11 | ((quasiexperimental or quasi-experimental) adj3 (study or studies or trial*)).ti,ab,hw. | 169 |
| 12 | (phase adj3 (III or "3") adj3 (study or studies or trial*)).ti,ab. | 8 |
| 13 | 1 or 2 or 3 or 4 or 5 or 6 or 7 or 8 or 9 or 10 or 11 or 12 | 7208 |
| 14 | (cohort adj (study or studies)).tw. | 870 |
| 15 | Cohort analy$.tw. | 18 |
| 16 | (Follow up adj (study or studies)).tw. | 400 |
| 17 | (observational adj (study or studies)).tw. | 258 |
| 18 | Longitudinal.tw. | 4932 |
| 19 | Retrospective.tw. | 1005 |
| 20 | Or/14-19 | 7103 |
| 21 | (systematic adj3 review$).ti,ab. | 3020 |
| 22 | 13 or 20 or 21 | 16158 |
| 23 | [Social Mobility/] | 0 |
| 24 | (Social$ adj3 (mobil$ or change)).ti,ab. | 1840 |
| 25 | ((absolute or relative or "long range" or "short range") adj3 mobil$).ti,ab. | 10 |
| 26 | [exp Socioeconomic Status/ and mobil$.ti,ab.] | 0 |
| 27 | (Economic Status and (mobil$ or change)).ti,ab. | 54 |
| 28 | ((socioeconomic or socio-economic or economic$ or income or salary or salaries or earn or earning or wealth or occupation or employment or job) adj3 (mobil$ or change)).ti,ab. | 621 |
| 29 | ((intergenerational or inter-generational Intragenerational or Intra-generational) adj2 (mobil$ or change)).ti,ab. | 31 |
| 30 | ((low or medium or high) adj3 depriv$).ti,ab. | 160 |
| 31 | ((father$ or mother$ or parent$ or grandfather$ or grandmother$ or early-life) adj3 (occupation* or employment$ or job or education or upbringing)).ti,ab. | 1182 |
| 32 | [*Social Class/] | 0 |
| 33 | ((social class or social background) adj3 (mobil$ or change)).ti,ab. | 12 |
| 34 | ((poor or disadvantage$) adj3 (background or mobil$)).ti,ab. | 70 |
| 35 | [*Education/ and mobil$.ti,ab.] | 0 |
| 36 | ((education$ and (attainment or performance or results or outcome$ or grade$)) adj3 mobil$).ti,ab. | 75 |
| 37 | Or/23-36 | 3799 |
| 38 | [exp Mental Disorders/] | 0 |
| 39 | (mental$ adj3 (health$ or ill$ or unwell$ or disorder$)).ti,ab. | 28881 |
| 40 | 38 or 39 | 28881 |
| 41 | 22 and 37 and 40 | 27 |

**Database:** Health Management Information Consortium (HMIC)

**Host:** OVID

**Search parameters:** 1979 to May 2019

**Date of search:** Monday September 30^th^ 2019

Search Strategy:

| **#** | **Searches** | **Results** |
| --- | --- | --- |
| 1 | (random* or sham or placebo*).ti,ab,hw. | 11696 |
| 2 | ((singl* or doubl*) adj (blind* or dumm* or mask*)).ti,ab,hw. | 536 |
| 3 | ((tripl* or trebl*) adj (blind* or dumm* or mask*)).ti,ab,hw. | 1 |
| 4 | (control* adj3 (study or studies or trial* or group*)).ti,ab. | 8161 |
| 5 | (Nonrandom* or non random* or non-random* or quasi-random* or quasirandom*).ti,ab,hw. | 339 |
| 6 | allocated.ti,ab,hw. | 1595 |
| 7 | ((open label or open-label) adj5 (study or studies or trial*)).ti,ab,hw. | 88 |
| 8 | ((equivalence or superiority or non-inferiority or noninferiority) adj3 (study or studies or trial*)).ti,ab,hw. | 62 |
| 9 | (pragmatic study or pragmatic studies).ti,ab,hw. | 12 |
| 10 | ((pragmatic or practical) adj3 trial*).ti,ab,hw. | 211 |
| 11 | ((quasiexperimental or quasi-experimental) adj3 (study or studies or trial*)).ti,ab,hw. | 157 |
| 12 | (phase adj3 (III or "3") adj3 (study or studies or trial*)).ti,ab. | 59 |
| 13 | 1 or 2 or 3 or 4 or 5 or 6 or 7 or 8 or 9 or 10 or 11 or 12 | 15629 |
| 14 | (cohort adj (study or studies)).tw. | 3389 |
| 15 | Cohort analy$.tw. | 143 |
| 16 | (Follow up adj (study or studies)).tw. | 640 |
| 17 | (observational adj (study or studies)).tw. | 1444 |
| 18 | Longitudinal.tw. | 3275 |
| 19 | Retrospective.tw. | 3170 |
| 20 | 14 or 15 or 16 or 17 or 18 or 19 | 10566 |
| 21 | (systematic adj3 review$).ti,ab. | 4400 |
| 22 | 13 or 20 or 21 | 27258 |
| 23 | Social Mobility/ | 41 |
| 24 | (Social$ adj3 (mobil$ or change)).ti,ab. | 489 |
| 25 | ((absolute or relative or "long range" or "short range") adj3 mobil$).ti,ab. | 2 |
| 26 | exp Socioeconomic Status/ and mobil$.ti,ab. | 37 |
| 27 | (Economic Status and (mobil$ or change)).ti,ab. | 54 |
| 28 | ((socioeconomic or socio-economic or economic$ or income or salary or salaries or earn or earning or wealth or occupation or employment or job) adj3 (mobil$ or change)).ti,ab. | 268 |
| 29 | ((intergenerational or inter-generational Intragenerational or Intra-generational) adj2 (mobil$ or change)).ti,ab. | 11 |
| 30 | ((low or medium or high) adj3 depriv$).ti,ab. | 155 |
| 31 | ((father$ or mother$ or parent$ or grandfather$ or grandmother$ or early-life) adj3 (occupation* or employment$ or job or education or upbringing)).ti,ab. | 493 |
| 32 | *Social Class/ | 0 |
| 33 | ((social class or social background) adj3 (mobil$ or change)).ti,ab. | 11 |
| 34 | ((poor or disadvantage$) adj3 (background or mobil$)).ti,ab. | 42 |
| 35 | *Education/ and mobil$.ti,ab. | 0 |
| 36 | ((education$ and (attainment or performance or results or outcome$ or grade$)) adj3 mobil$).ti,ab. | 47 |
| 37 | Or/23-36 | 1499 |
| 38 | exp Mental Disorders/ | 23585 |
| 39 | (mental$ adj3 (health$ or ill$ or unwell$ or disorder$)).ti,ab. | 21893 |
| 40 | 38 or 39 | 37147 |
| 41 | 22 and 37 and 40 | 52 |

**Database:** ASSIA

**Host:** Pro Quest

**Search parameters:** 1987 – Current

**Date of search:** Wednesday October 2^nd^ 2019

Search Strategy

Set#: S1

Searched for: MAINSUBJECT.EXACT.EXPLODE("Social mobility")

Databases: Applied Social Sciences Index & Abstracts (ASSIA)

Results: 458

Set#: S2

Searched for: ab((Social* NEAR/2 (mobil* or change)) ) OR ti((Social* NEAR/2 (mobil* or change)) )

Databases: Applied Social Sciences Index & Abstracts (ASSIA)

Results: 6097

Set#: S3

Searched for: ab(((absolute or relative or "long range" or "short range") NEAR/2 mobil*) ) OR ti(((absolute or relative or "long range" or "short range") NEAR/2 mobil*) )

Databases: Applied Social Sciences Index & Abstracts (ASSIA)

Results: 43

Set#: S4

Searched for: ab((Economic Status and (mobil$ or change)) ) OR ti((Economic Status and (mobil$ or change)) )

Databases: Applied Social Sciences Index & Abstracts (ASSIA)

Results: 634

Set#: S5

Searched for: ab(((socioeconomic or socio-economic or economic* or income or salary or salaries or earn or earning or wealth or occupation or employment or job) NEAR/2 (mobil* or change)) ) OR ti(((socioeconomic or socio-economic or economic* or income or salary or salaries or earn or earning or wealth or occupation or employment or job) NEAR/2 (mobil* or change)) )

Databases: Applied Social Sciences Index & Abstracts (ASSIA)

Results: 2970

Set#: S6

Searched for: ab(((low or medium or high) NEAR/2 depriv*) ) OR ti(((low or medium or high) NEAR/2 depriv*) )

Databases: Applied Social Sciences Index & Abstracts (ASSIA)

Results: 284

Set#: S7

Searched for: ab(((father* or mother* or parent* or grandfather* or grandmother* or early-life) NEAR/2 (occupation* or employment* or job or education or upbringing)) ) OR ti(((father* or mother* or parent* or grandfather* or grandmother* or early-life) NEAR/2 (occupation* or employment* or job or education or upbringing)) )

Databases: Applied Social Sciences Index & Abstracts (ASSIA)

Results: 3910

Set#: S8

Searched for: ab(((poor or disadvantage*) NEAR/2 (background or mobil*)) ) OR ti(((poor or disadvantage*) NEAR/2 (background or mobil*)) )

Databases: Applied Social Sciences Index & Abstracts (ASSIA)

Results: 440

Set#: S9

Searched for: ab(((intergenerational or inter-generational Intragenerational or Intra-generational) AND (mobil* or change)) ) OR ti(((intergenerational or inter-generational Intragenerational or Intra-generational) AND (mobil* or change)) )

Databases: Applied Social Sciences Index & Abstracts (ASSIA)

Results: 442

Set#: S10

Searched for: ab(((social class or social background) AND (mobil* or change)) ) OR ti(((social class or social background) AND (mobil* or change)) )

Databases: Applied Social Sciences Index & Abstracts (ASSIA)

Results: 2900

Set#: S11

Searched for: ab((social class OR social background) AND (mobil* OR change)) OR ti((social class OR social background) AND (mobil* OR change))

Databases: Applied Social Sciences Index & Abstracts (ASSIA)

Results: 2900

Set#: S12

Searched for: S1 or S2 or S3 or S4 or S5 or S6 or S7 or S8 or S9 or S10 or S11

Databases: Applied Social Sciences Index & Abstracts (ASSIA)

These databases are searched for part of your query.

Results: 16208

Set#: S13

Searched for: MAINSUBJECT.EXACT.EXPLODE("Mental health")

Databases: Applied Social Sciences Index & Abstracts (ASSIA)

Results: 27727

Set#: S14

Searched for: ti((mental* NEAR/2 (health* or ill* or unwell* or disorder*))) AND ab((mental* NEAR/2 (health* or ill* or unwell* or disorder*)))

Databases: Applied Social Sciences Index & Abstracts (ASSIA)

Results: 22664

Set#: S15

Searched for: S13 or S14

Databases: Applied Social Sciences Index & Abstracts (ASSIA)

These databases are searched for part of your query.

Results: 9659

Set#: S16

Searched for: S12 and S15

Databases: Applied Social Sciences Index & Abstracts (ASSIA)

These databases are searched for part of your query.

Results: 154

Set#: S17

Searched for: ti((random* or control* or trial or cohort or longitudinal or prospective or retrospective or "follow up") ) AND ab((random* or control* or trial or cohort or longitudinal or prospective or retrospective or "follow up") )

Databases: Applied Social Sciences Index & Abstracts (ASSIA)

Results: 45851

Set#: S18

Searched for: S16 and S17

Databases: Applied Social Sciences Index & Abstracts (ASSIA)

Results: 16

**Database:** ERIC

**Host:** Pro Quest

**Search parameters:** 1966 – Current

**Date of search:** Wednesday October 2^nd^ 2019

Search Strategy

Set#: S1

Searched for: MAINSUBJECT.EXACT.EXPLODE("Social Mobility")

Databases: ERIC

Results: 1587

Set#: S2

Searched for: ab((Social* NEAR/2 (mobil* or change)) ) OR ti((Social* NEAR/2 (mobil* or change)) )

Databases: ERIC

Results: 9784

Set#: S3

Searched for: ab(((absolute or relative or "long range" or "short range") NEAR/2 mobil*) ) OR ti(((absolute or relative or "long range" or "short range") NEAR/2 mobil*) )

Databases: ERIC

Results: 29

Set#: S4

Searched for: ab((Economic Status and (mobil$ or change)) ) OR ti((Economic Status and (mobil$ or change)) )

Databases: ERIC

Results: 929

Set#: S5

Searched for: ab(((socioeconomic or socio-economic or economic* or income or salary or salaries or earn or earning or wealth or occupation or employment or job) NEAR/2 (mobil* or change)) ) OR ti(((socioeconomic or socio-economic or economic* or income or salary or salaries or earn or earning or wealth or occupation or employment or job) NEAR/2 (mobil* or change)) )

Databases: ERIC

Results: 4330

Set#: S6

Searched for: ab(((low or medium or high) NEAR/2 depriv*) ) OR ti(((low or medium or high) NEAR/2 depriv*) )

Databases: ERIC

Results: 100

Set#: S7

Searched for: ab(((father* or mother* or parent* or grandfather* or grandmother* or early-life) NEAR/2 (occupation* or employment* or job or education or upbringing)) ) OR ti(((father* or mother* or parent* or grandfather* or grandmother* or early-life) NEAR/2 (occupation* or employment* or job or education or upbringing)) )

Databases: ERIC

Results: 10404

Set#: S8

Searched for: ab(((poor or disadvantage*) NEAR/2 (background or mobil*)) ) OR ti(((poor or disadvantage*) NEAR/2 (background or mobil*)) )

Databases: ERIC

Results: 889

Set#: S9

Searched for: ab(((intergenerational or inter-generational Intragenerational or Intra-generational) AND (mobil* or change)) ) OR ti(((intergenerational or inter-generational Intragenerational or Intra-generational) AND (mobil* or change)) )

Databases: ERIC

Results: 439

Set#: S10

Searched for: ab(((social class or social background) AND (mobil* or change)) ) OR ti(((social class or social background) AND (mobil* or change)) )

Databases: ERIC

Results: 4744

Set#: S11

Searched for: ab((social class OR social background) AND (mobil* OR change)) OR ti((social class OR social background) AND (mobil* OR change))

Databases: ERIC

Results: 4744

Set#: S12

Searched for: S1 or S2 or S3 or S4 or S5 or S6 or S7 or S8 or S9 or S10 or S11

Databases: ERIC

These databases are searched for part of your query.

Results: 29603

Set#: S13

Searched for: MAINSUBJECT.EXACT.EXPLODE("Mental Health")

Databases: ERIC

Results: 11455

Set#: S14

Searched for: ti((mental* NEAR/2 (health* or ill* or unwell* or disorder*))) AND ab((mental* NEAR/2 (health* or ill* or unwell* or disorder*)))

Databases: ERIC

Results: 4724

Set#: S15

Searched for: S13 or S14

Databases: ERIC

These databases are searched for part of your query.

Results: 13157

Set#: S16

Searched for: S12 and S15

Databases: ERIC

These databases are searched for part of your query.

Results: 261

Set#: S17

Searched for: ti((random* or control* or trial or cohort or longitudinal or prospective or retrospective or "follow up") ) AND ab((random* or control* or trial or cohort or longitudinal or prospective or retrospective or "follow up") )

Databases: ERIC

Results: 20898

Set#: S18

Searched for: S16 and S17

Databases: ERIC

Results: 6

----------------------------------------------------------

WEB SEARCHES

| Date searched | Search interface and edition and URL | Search Terms | Notes |
| --- | --- | --- | --- |
| 30/09/2019 | Google https://www.google.com/ | ((social mobility) and (mental health)) | Yield one page of results (n=10 records), 10 records checked all EX1 |
| 30/09/2019 | Google https://www.google.com/ | ((social class) and (mental health)) | Yield one page of results (n=7 records), 7 records checked all EX2 |
| 30/09/2019 | Google https://www.google.com/ | ((intergenerational) and (social mobility) and (mental health)) | Yield one page of results (n=4 records), 4 records checked all EX2 |
| 30/09/2019 | Google https://www.google.com/ | ((intergenerational) and (social class) and (mental health)) | Yield one page of results (n=6 records), 6 records checked all EX2 |
| 01/10/2019 | Google https://www.google.com/ | ((intragenerational) and (social mobility) and (mental health)) | Yield one page of results (n=6 records), 6 records checked all EX0 |
| 01/10/2019 | Google https://www.google.com/ | ((intragenerational) and (social class) and (mental health)) | Yield one page of results (n=12 records), 12 records checked all EX3 |
| 01/10/2019 | Dogpile https://www.dogpile.com/ | ((social mobility) and (mental health)) | Yield five page of results, checked 3 pages as studies were repeating (n=25 records), 25 records checked all EX18 |
| 01-02/10/2019 | Dogpile https://www.dogpile.com/ | (effects of (mental health) on(intergenerational social mobility)) | Yield eight page of results, checked 7 pages as studies were repeating (n=53 records), 53 records checked all EX43 |
| 02/10/2019 | Google  https://www.google.com/ | (the effects of (mental illness) on (intergenerational social mobility)) | Yield three page of results, cheked pages (n=26 records), 26 records checked all EX20 |

References

1. Cooper C, Dawson S, Peters J, Varley-Campbell J, Cockcroft E, Hendon J, et al. Revisiting the need for a literature search narrative: A brief methodological note. Research synthesis methods. 2018;9(3):361-5.

2. Lefebvre C, Manheimer E, Glanville J. Chapter 6: Searching for studies. 2011 [cited Accessed 7th December 2017]. In: Cochrane Handbook for Systematic Reviews of Interventions [Internet]. The Cochrane Collaboration, [cited Accessed 7th December 2017]. Available from: <http://handbook.cochrane.org/>.

3. DRAFT REPORT ON FOSTERING SOCIAL MOBILITY AS A CONTRIBUTION TO SOCIAL COHESION,. Strasbourg: EUROPEAN COMMITTEE OF SOCIAL COHESION (CDCS),; 2011.

4. Social mobility indicators Gov.Uk: The Deputy Prime Minister’s Office; 2015 [Available from: <https://www.gov.uk/government/publications/social-mobility-indicators/social-mobility-indicators>.

5. Department for business invovation and skils. Social Mobility: A literature review. London: Department for business invovation and skils,; 2011.

6. Elhakeem A, Hardy R, Bann D, Caleyachetty R, Cosco TD, Hayhoe RP, et al. Intergenerational social mobility and leisure-time physical activity in adulthood: a systematic review. Journal of Epidemiology and Community Health. 2017;71(7):673-80.

7. Group BC. THE STATE OF SOCIAL MOBILITY IN THE UK. UK: Sutton Trust; 2017.

8. Motta JVdS, Lima NP, Olinto MTA, Gigante DP. Social mobility and smoking: a systematic review. Ciência & Saúde Coletiva. 2015;20:1515-20.

9. Torche F. How do we characteristically measure and analyze intergenerational mobility? 2013.

10. de Quadros Lde C, Laura HC, Quevedo Lde A, Gigante DP. [Effects of social mobility on adult mental health:a systematic review of the literature]. Cien Saude Colet. 2016;21(2):443-8.

11. Finegan M, Firth N, Wojnarowski C, Delgadillo J. Associations between socioeconomic status and psychological therapy outcomes: A systematic review and meta-analysis. Depression and anxiety. 2018;35(6):560-73.

12. Hoven H, Siegrist J, Goldberg M, Ribet C, Zins M, Wahrendorf M. Intragenerational social mobility and depressive symptoms. Results from the French CONSTANCES cohort study. SSM - Population Health. 2019;7:100351.

13. The Canadian Agency for Drugs and Technologies in Health (CADTH). Strings Attached: CADTH’s Database Search FIlters 2018 [Available from: <https://www.cadth.ca/resources/finding-evidence/strings-attached-cadths-database-search-filters#guide>.

14. Cooper C, Varley-Campbell J, Carter P. Established search filters may miss studies when identifying randomised controlled trials. Journal of Clinical Epidemiology. 2019;112(August):12-9.

15. Healthcare Improvement Scotland: SIGN. Search Filters Edinburgh: Healthcare Improvement Scotland: SIGN; 2019 [cited 2019 Jul 19]. Available from: <https://www.sign.ac.uk/search-filters.html>.
